# Chelerythrine down regulates expression of VEGFA, BCL2 and KRAS by arresting G-Quadruplex structures at their promoter regions

**DOI:** 10.1038/srep40706

**Published:** 2017-01-19

**Authors:** Jagannath Jana, Soma Mondal, Payel Bhattacharjee, Pallabi Sengupta, Tanaya Roychowdhury, Pranay Saha, Pallob Kundu, Subhrangsu Chatterjee

**Affiliations:** 1 Department of Biophysics, Bose Institute, Kolkata, WB, India; 2 Division of Plant Biology, Bose Institute, Kolkata, WB, India; 3 Cancer Biology and Inflammatory Disorder Division, Kolkata, WB, India

## Abstract

A putative anticancer plant alkaloid, Chelerythrine binds to G-quadruplexes at promoters of VEGFA, BCL2 and KRAS genes and down regulates their expression. The association of Chelerythrine to G-quadruplex at the promoters of these oncogenes were monitored using UV absorption spectroscopy, fluorescence anisotropy, circular dichroism spectroscopy, CD melting, isothermal titration calorimetry, molecular dynamics simulation and quantitative RT-PCR technique. The pronounced hypochromism accompanied by red shifts in UV absorption spectroscopy in conjunction with ethidium bromide displacement assay indicates end stacking mode of interaction of Chelerythrine with the corresponding G-quadruplex structures. An increase in fluorescence anisotropy and CD melting temperature of Chelerythrine-quadruplex complex revealed the formation of stable Chelerythrine-quadruplex complex. Isothermal titration calorimetry data confirmed that Chelerythrine-quadruplex complex formation is thermodynamically favourable. Results of quantative RT-PCR experiment in combination with luciferase assay showed that Chelerythrine treatment to MCF7 breast cancer cells effectively down regulated transcript level of all three genes, suggesting that Chelerythrine efficiently binds to *in cellulo* quadruplex motifs. MD simulation provides the molecular picture showing interaction between Chelerythrine and G-quadruplex. Binding of Chelerythrine with BCL2, VEGFA and KRAS genes involved in evasion, angiogenesis and self sufficiency of cancer cells provides a new insight for the development of future therapeutics against cancer.

DNA can adopt right handed double helical B-form structure^1^. Under certain conditions DNA can adopt an array of non B-form unique unusual secondary structures based on particular sequence motif^2^. These unusual secondary structures may have an effect on a number of biologically significant processes^3^. G-quartets are composed of square planer arrangement of four Guanine bases stabilised by eight Hoogsteen hydrogen bonds (N1-O6 & N2-N7). The O6 atoms of Guanine surrounding the central core form co-ordination bond with monovalent cations (K^+^, Na^+^, NH_4_^+^ etc.) to minimize electrostatic interaction. G-quadruplex can adopt parallel, anti-parallel and hybrid structures depending upon their strand orientation, type of cation and sequence being used^4^.

Quadruplex may exist in thousand of gene promoters influencing their expression^5^ has been proposed that G-quadruplexes may be directly implicated in gene regulation at the level of transcription^5^,^6^. Six important proceedings in the malignant process are evasion of apoptosis, insensitivity to anti-growth signals, self-sufficiency in growth signals, sustained angiogenesis, limitless replicative potential and tissue invasion and metastasis. These are known as six hallmarks of cancer^7^. These important events are linked with G-quadruplex forming gene promoters including c-MYC^8^, c-KIT^9^ and KRAS^10^ (self-sufficiency), RB1 (insensitivity), BCL2^11^ (evasion of apoptosis), VEGFA (angiogenesis)^12^ hTERT (limitless replication)^13^ and PDGFA (metastasis). G-quadruplex structures in oncogenic promoters are indicators of the six hallmarks of cancer. These oncogene promoters form G-quadruplexes with immense variety in their folding patterns. Stabilization of G-quadruplexes are directly involved in the regulation of gene expression which blocks the binding of a repressor protein and functions as an activator of transcription, making them attractive anticancer drug target.

Several studies suggested that formation and stabilization of G-quadruplex in the promoter region of proto oncogene like c-MYC^3^, c-KIT^14^, KRAS^15^, BCL2^16^, VEGFA^17^ etc. affects at the transcription level. BCL2, KRAS and VEGFA, most frequently mutated oncogenes in human cancer are overexpressed in different types of human malignancies. The B-cell CLL/lymphoma 2 (BCL2)^18^ gene plays an important role in cell survival functioning as an inhibitor of cell apoptosis being connected with anti-apoptotic proteins. BCL2 has been found to be overexpressed in a wide range of human tumors, including prostate^19^, breast^20^ colorectal^21^, cervical and non-small cell lung carcinomas and B-cell and T-cell lymphomas. Vascular endothelial growth factor A (VEGFA) is angiogenic growth factor that plays an essential role in angiogenesis and tumor progression^22^. HRAS, KRAS and NRAS are RAS genes which serve as switches between the epidermal growth factor receptor (EGFR) and the nucleus that influence cell growth and apoptosis. KRAS is one of the most frequently mutated oncogenes in different types of human cancers including colorectal^23^, pancreatic^24^ and lung cancers^25^. Several approaches to transcriptional repression of oncogenes at promoter level have been studied by small molecules yet remaining undruggable.

To date, a number of G-quadruplex-stabilizing ligands have been characterized^26^. Only a few of the G-quadruplex-stabilizing agents have been victorious in a range of clinical trials^27^. G-quartets have large π-surfaces and hence tend to stack on each other due to π-π stacking^28^. Most of these small molecules like TmPyP4, telomestatin, berberine, sanguinarine etc.^29^ have large planer π-aromatic surface responsible for the stacking interaction with G-quadruplex^30^. Chelerythrine (Fig. 1A), a benzophenanthridine plant alkaloid acts as inhibitor of protein kinase C with potential anticancer activities^31^. Earlier reports showed that Chelerythrine induced apoptosis in several cancer cell lines^32^. Chelerythrine exhibited broad spectrum of cytotoxic activity against different types of cancer cell with minimal toxicity^33^. It was previously reported that Chelerythrine was recognized by human telomeric DNA and RNA G-quadruplex along with c-KIT promoter^9^,^34^. In this study for the first time we have shown Chelerythrine down regulates the expression of BCL2, VEGFA and KRAS genes.

Here in this report we have studied the binding of Chelerythrine with G-quadruplex forming promoter sequences of BCL2, VEGFA and KRAS. The various biophysical techniques such as UV absorption spectroscopy, fluorescence anisotropy, circular dichroism spectroscopy (CD), CD melting and isothermal titration calorimetry (ITC) in conjunction with molecular dynamics simulation confirm interaction and stabilization of Chelerythrine-quadruplex complex. Quantitative RT-PCR experiment and luciferase assay were conducted to show the down regulation of expression of these three genes upon treatment with Chelerythrine.

## Methods and Materials

### Preparation of Sample

Commercially synthesized oligonucleotides were purchased from Eurofins Genomics India Pvt. Ltd. The following three oligonucleotide sequences were selected in this study.

**VEGFA:** 5′-GGGGGCGGGCCGGGGGCGGGGTCCCGGCGGGGCGGAG-3′

**BCL2:** 5′-CGGGCGCGGGAGGAATTGGGCGGGAGC-3′

**KRAS:** 5′-GGGAGGGAGGGAAGGAGGGAGGGAGGGA-3′

The DNA sequences, 5′-AGGGTGGGGAGGGT-3′ and 5′-ACCCTCCCCACCCT-3′ were used for duplex. Potassium phosphate monobasic, potassium phosphate dibasic, potassium chloride, Chelerythrine were purchased from Sigma Aldrich. All experiments were carried out using 10 mM potassium phosphate buffer containing 100 mM potassium chloride at pH 7.0. Chelerythrine were dissolved in the same buffer. All oligonucleotides were dissolved in the above mentioned buffer and heated in water bath at 95 °C for 10 minutes. The oligonucleotides were slowly cooled to room temperature and then stored at 4 °C for 24 hours.

MCF7 cells obtained from human breast cancer adenocarcinoma cells were maintained in cell culture media consisting of Dulbecco’s modified Eagle’s medium (DMEM, high glucose, with L-glutamine) (Invitrogen) supplemented with10% fetal bovine serum, 50 U/ml penicillin, 50 μg/ml streptomycin, and under 5% CO_2_ air mixture at 37 °C.

### UV spectroscopy

All UV absorption spectra were measured in Hitachi U-2910 spectrophotometer using a quartz cuvette with 1 cm path length. The spectra were scanned from 300 to 600 nm. 10 μM of Chelerythrine were titrated against increasing concentration (0–20 μM) of VEGFA, BCL2 and KRAS until saturation was almost reached. The changes in absorption of Chelerythrine were measured upon addition of quadruplexes. The UV titration data were also used to calculate the dissociation constant (K_D_) value by plotting the change in absorbance (∆A/∆Amax) at 316 nm of Chelerythrine versus increasing concentration of VEGFA, KRAS and BCL2. The experimental data points obtained were fitted in one site saturation binding equation.


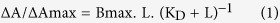


where ∆A/∆Amax = Change in absorbance, L = quadruplex concentration and K_D_ = dissociation constant.

Similar experiment was performed with duplex DNA where 10 μM Chelerythrine was titrated with increasing concentrations of duplex DNA (0–20 μM) under similar experimental condition.

### Fluorescence spectroscopy

Fluorescence titration was performed using Hitachi spectrophotometer (F-7000 FL spectrophotometer). Fluorescence spectra were recorded using a 1 cm cuvette. The excitation wavelength was fixed at 316 nm and the emission wavelength was scanned from 360 nm to 600 nm. To determine the mode of binding of Chelerythrine with quadruplex sequences ethidium bromide displacement assay were performed using fluorescence spectroscopy. Initially 5 μM ethidium bromide was saturated with 10 μM quadruplex and then titrated with increasing concentration of Chelerythrine. The excitation wavelength of ethidium bromide was set at 510 nm and emission profile was monitored at a range of 520–640 nm.

### Fluorescence anisotropy

Steady-state anisotropy was recorded with Hitachi model F-7000 FL spectrometer equipped with a polarized accessory. Anisotropy values were calculated based upon the fluorescence property of Chelerythrine. The fluorescence anisotropy (r) values were obtained using the expression equation:


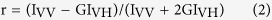



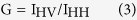


where I_VV_ and I_VH_ are emmision intensities of the parallel and perpendicularly polarized light at 410 nm respectively. G is correction factor. The slits for excitation and emission were set at 5 nm. 10 μM Chelerythrine was titrated with increasing concentrations of VEGFA, BCL2 and KRAS. Equilibrium dissociation constant (K_D_) were determined by fitting the anisotropy data using the following equation^35^:


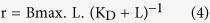


where r = fluorescence anisotropy, L = quadruplex concentration and K_D_ = dissociation constant.

To determine, the binding of Chelerythrine to duplex DNA steady state anisotropy was measured. 10 μM Chelerythrine was titrated with increasing concentrations of duplex DNA (0–20 μM) under similar experimental condition.

### CD spectroscopy

Circular dichroism spectra were recorded on a Jasco 815 spectrometer at 25 °C. The CD spectra were recorded within a wavelength range 320 nm to 210 nm with a scan speed of 100 nm/min and step size of 1 nm. The band width was 1 nm. All measurements were carried out using a 1 mm path length cuvette in a reaction volume 350 μL. 10 μM of each quadruplex sequences (BCL2, KRAS and VEGFA) were titrated with increasing concentration (10 μM, 20 μM and 30 μM) of Chelerythrine upto a ratio of 1:3. Readings were noted 5 mins after each addition to ensure complete complex formation. DNA melting experiments were performed using the same cuvette and reaction mixture with the temperature being varied from 10 °C to 90 °C at an interval of 5 °C. 1:2 ratio of quadruplex-Chelerythrine (quadruplex: Chelerythrine = 1:2) complex were used for the CD melting experiment. All the CD melting experiments were repeated twice.

### Isothermal titration calorimetry (ITC)

ITC were carried out to determine thermodynamics of binding of Chelerythrine with quadruplex sequences using a VP ITC Micro calorimeter at 25 °C. A samples cell containing 10 μM Chelerythrine were titrated against 100 μM quadruplex sequences. Samples were extensively degassed prior to titration. Total no. of injection was 20 at an interval of 150 seconds with 2 μL quadruplex per injection. A blank experiment was performed in which quadruplex sequences were injected into buffer (10 mM potassium phosphate buffer containing 100 mM potassium chloride at pH 7.0.) with no Chelerythrine to correct the data due to dilution. The heat of interaction was calculated after subtraction from heat of dilution. The corrected values were plotted against molar ratio. The raw data were analysed using Origin software provided with the instrument. The data were fitted using one set of site model. Equilibrium association constant (K_A_), change in enthalpy of reaction (∆H), change in Gibb’s free energy (∆G) and entropy (∆S) were measured using the following equation:


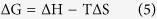


The thermodynamics of binding of Chelerythrine binding to GC rich duplex DNA was also measured. 10 μM of Chelerythrine was titrated with 100 μM of duplex DNA at 25 °C keeping others parameters identical with those of Chelerythrine binding to quadruplex sequences. A blank experiment was performed where duplex DNA was injected in free buffer solution. The heat change obtained from Chelerythrine-duplex DNA binding was subtracted from blank experiment. The data points obtained were fitted into ‘one set of site’ binding model.

### Treatment, RNA isolation and cDNA preparation

Cell cultures were maintained in 60 mm culture plates until they reached 60% confluence. Cells were then treated with 15 nM and 150 nM Chelerythrine. The control set received equivalent amount of water, as the drug was suspended in water. Treated cells were maintained for 24 hours, after which they were observed under microscope to ensure that the cells were still alive, and live cells were taken for RNA isolation. RNA was prepared from control and treated cell cultures using TRIzol^®^ reagent (Invitrogen) following manufacturer’s protocol. The concentration of isolated RNA was determined by measuring absorbance at 260 nm in a spectrophotometer and the quality and integrity of RNA was checked by electrophoresis of denatured RNA in a 1.5% Agarose gel (Figure S2A). cDNA was prepared from RNA samples which showed distinct 28 S and 18 S bands (2:1 intensity ratio) in the gel. Total RNA (3 μg, from both control and treated) was reverse-transcribed using 200 U RevertAid reverse transcriptase (Fermentas) and random hexamer primers (5 μM) in 20 μl reaction volume at 42 °C, following manufacturer’s protocol.

### Primer design

Sequence information of all genes were acquired from the Human **–** Ensembl website. At least one of the primers was designed from an intron-exon junction of a gene to avoid any non specific amplification from genomic DNA contamination (if any). IDT Oligo Analyzer was utilized to determine the annealing temperature, homo or hetero dimer formation tendency and specificity of the designed primers. Amplification of a single product of expected size with a primer pair confirmed their specificity in a reaction (Figure S2B). Information about the primers used in this study is summarized in Table S5.

### Real Time PCR (RT-PCR)

The relative expression level of the target genes was analyzed by performing quantitative real time PCR. Real time PCR reactions were performed with only 5% of cDNA reactions using Power SYBR^®^ Green PCR mix reagent and Applied Biosystems 7500 FAST system. The thermal cycling conditions were as follows: an initial denaturation step by heating at 95 °C for 10 minutes followed by 40 cycles of 30 seconds initial denaturation (at 95 °C), 30 seconds in appropriate melting temperature according to the primers and 45 seconds of extension in 72 °C. Controls included PCRs performed with a mock cDNA (reaction without reverse transcriptase) and no-template control (H_2_O control) (Figure S2C). Specific products were detected as clear single peaks at their melting temperature in the fluorescence versus temperature plot (melting curve). Representative PCR products were resolved in a 2.5% Agarose gel to confirm the presence of a single band of expected size. The relative expression level of the gene of interest was determined after normalizing the data by GAPDH expression level and was calculated for each sample using the 2^−ΔΔcT^ method^36^. All reactions were done at least in duplicate using three independent RNA preparations.

### Construction of reporter luciferase vectors

The promoter sequences of BCL2, VEGFA, and KRAS with or without the upstream quadruplex-forming elements were cloned into pGL4.72 [*hRlucCP*] luciferase vectors at KpnI and HindIII restriction sites. The G-Quadruplex(GQ)-enriched promoter sequences were PCR (Polymerase chain reaction) amplified from the genomic DNA, isolated from MCF-7 cell line using wild-type (wt) primers (Promoter+GQ), which contain KpnI and HindIII restriction sites (Table S6). The PCR products were purified using PureLink^®^ PCR Purification Kit (Invitrogen Catalog no. K310001), cut with KpnI and HindIII enzymes and cloned into same restriction sites within pGL4.72 [*hRlucCP*] luciferase vector. The GQ-deficient constructs (GQ-null) were further created over respective wild-type templates (Promoter+GQ) *via* overlap-extension PCR techniques using mutagenic primers (Table S6). In short, the first round PCR reaction was carried out using the mutagenic (mut) primers and all the PCR products are purified and combined to use as templates for the final extension using wild-type (wt) primers. The resultant products were cleaned, cleaved by restriction enzymes and cloned as explained above. Successful cloning of different inserts was validated by sequencing from Biobharati India Pvt. Ltd (Sequence report Supplementary others). The details of primer sequences for the construction of reporter plasmids were provided in the Table S6.

### Cell culture and transfection

MCF-7 cells were sub-cultured into 24-well microtiter plates at a density of 2.5 × 10^4^ cells/well. The ‘GQ null’ and ‘Promoter+GQ’ constructs were transformed into competent *E. coli* DH5α cells to amplify the the plasmids and the transformed cells were spread over LB (Luria broth) agar plates having 150 μg/ml Ampicillin and incubated at 37 °C overnight. The singular colony was picked from each plate and further grown into LB media containing Ampicillin for 12–16 hours at 37 °C incubator and shaker (200 rpm). Plasmids were isolated using QIAGEN Plasmid Midi Kit (Catalog no. 12143) and subjected to transfection by Lipofectamine 2000 (Thermo-Fisher Scientific Catalog no. 11668027) as per manufacturer’s protocol and incubated at 37 °C temperature and 5% CO_2_ for 48 hours.

### Luciferase assays

After 24 hours of transfection, cells were treated with Chelerythrine at an increasing concentration gradient (15, 50, 75, 100 nM). Cell are taken out after 24 hours of treatment and washed with 1X PBS (Phosphate buffered saline), and scraped in ice with 1X PLB (Passive lysis buffer) and mixed with LAR II buffer (Luciferase assay reagent) as per manufacturer’s protocol (Promega Luciferase Assay System, E1500). Luminescence was measured in GloMax^®^ 20/20 Single-Tube Luminometer (Promega).

### Docking

Chelerythrine were docked with BCL2 and VEGFA in Glide module using standard precision (SP) mode (Glide, version 5.5, Schrodinger, Inc., New York, NY, 2009). The grid was prepared covering the entire structure of BCL2 and VEGFA with dimensions of 40×40×40 Å. First Chelerythrine were docked with BCL2 and VEGFA to obtain 1:1 complex. The second round of docking were performed using 1:1 complex to obtain 1:2 complex.

### Molecular dynamics simulation

MD simulations were performed in Amber14 using prmbsc0 modifications in conjunction with ff99SB force field for G-quadruplex^29^. Paramitization of Chelerythrine were carried out in simple harmonic function used by General Amber Force Field (GAFF) with AM1-BCC charge model^37^. In central core of quadruplex two K^+^ ions were added. The solvated systems were counter stabilized by K^+^ ions. K^+^ ions parameters were taken from Dang’s work^38^. TIP3P water model were used to solvate the system in an octahedral model with edge length extensions of 10 Å from solute atom. Simulation were performed using periodic boundary conditions with particle mesh Edward simulation method^39^. Lennard-Jones potentials and direct space interactions cutoff were 9 Å to correct long range vander-waals interactions. SHAKE algorithm were used for restraining hydrogen atoms with integration time step of 2 fs^40^. The energy minimization was carried out in explicit solvent conditions. MD simulations were continued upto 50 ns and the trajectory were collected at an interval of 2 ps for all system. The trajectory of MD simulation were analyzed using cpptraj module of Amber tools14^41^.

### Binding energy calculation

Free energy and binding energy were calculated using MM-PBSA method given in supporting information.

## Results

### Chelerythrine interacts with G-quadruplexes in VEGFA, BCL2 and KRAS using UV-absorption spectroscopy

**Figure 1:**
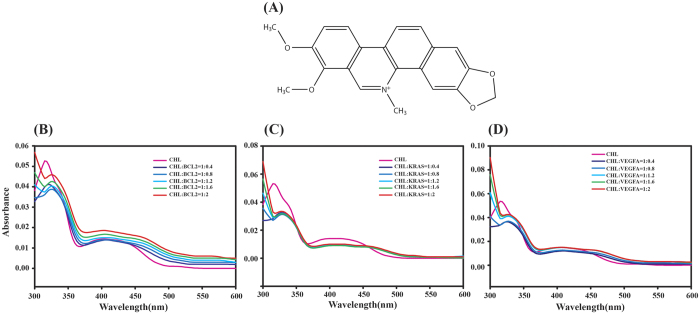
UV absorption spectra of Chelerythrine (10 μM) with increasing concentration of promoter sequences (BCL2, KRAS and VEGFA) showing red shift and hypochromism. (**B**) BCL2 (12 nm red shift, hypochromism 61%), (**C**) KRAS (14 nm red shift, hypochromism 50%), (**D**) VEGFA (12 nm red shift, hypochromism 63%). All experiments were carried out using 10 mM potassium phosphate buffer containing 100 mM potassium chloride at pH 7.0. (**A**) Chemical structure of Chelerythrine.

The binding of Chelerythrine with promoter sequences VEGFA, KRAS and BCL2 were studied using UV absorption spectroscopy. Chelerythrine showed an absorption band at 316 nm. In the absorption spectra of Chelerythrine we have seen hypochromism (50–63%) accompanied by red shift (12–14 nm) with increasing concentration of quadruplex sequences signifying strong complex formation (Table S1). The pronounced hypochromism accompanied by red shift is the outcome of π-π stacking interaction^42^. BCL2-Chelerythrine complex showed 60% hypochromism along with 12 nm red shift due to the stabilization of π* orbital of Chelerythrine upon complexation with BCL2 and thereby reducing their π-π* transition energy gap. Similar results have been observed for KRAS (Fig. 1C) and VEGFA (Fig. 1D). The presence of an isobestic point indicates the existence of a single type of Chelerythrine-quadruplex complex over the input concentration range of quadruplex (Fig. 1). The change in absorbance of Chelerythrine at 316 nm plotted as a function of increasing quadruplex concentration (Figure S1) to obtain dissociation constant (K_D_) (equation 1) for the Chelerythrine-quadruplex complexes are listed in Table S1. The binding affinity of Chelerythrine with quadruplex (promoter of VEGFA, BCL2 and KRAS) was found to be 4–16 fold higher compared to GC rich duplex DNA (Figure S3A).

### Binding of Chelerythrine with G-quadruplexes in VEGFA, BCL2 and KRAS by fluorescence anisotropy

**Figure 2:**
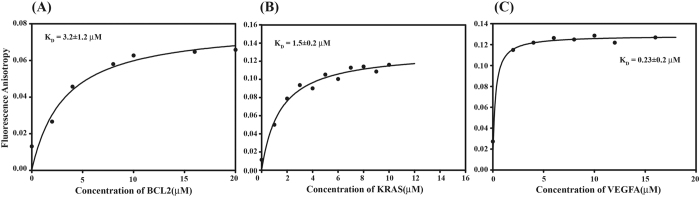
Fluorescence anisotropy of Chelerythrine (CHL) (10 μM) vs incresaing concentration of promoter sequences (BCL2, KRAS and VEGFA). (**A**) BCL2 (**B**) KRAS (**C**) VEGFA. All experiments were carried out using 10 mM potassium phosphate buffer containing 100 mM potassium chloride at pH 7.0.

The association of Chelerythrine with quadruplex sequences were investigated by an increase in fluorescence anisotropy of Chelerythrine upon addition of increasing quadruplex concentration. Binding of Chelerythrine with quadruplex reduces its mobility resulting in an increase of anisotropy of Chelerythrine in the bound form. Fluorescence anisotropy of Chelerythrine increases rapidly in the initial part of the curve upon addition of BCL2, VEGFA and KRAS (Fig. 2). Hence, we can conclude that Chelerythrine instantly binds to promoter sequences. Then small increase in fluroscence anisotropy and plateau indicates formation of the saturated Chelerythrine-quadruplex complex. The increase in fluorescence anisotropy of Chelerythrine complexed with BCL2, VEGFA and KRAS were found to be within 5 to 10 fold. The dissociation constant (K_D_), obtained by fitting the curve in ligand binding equation (equation 4) varies from 0.25 to 3.2 μM suggesting formation of strong Chelerythrine-quadruplex complex (Table S2). Chelerythrine showed higher affinity of binding to quadruplex structure of the promoter sequences of VEGFA, BCL2 and KRAS in comparison with GC rich duplex DNA (Figure S3B).

### Mode of binding of Chelerythrine with G-quadruplexes in VEGFA, BCL2 and KRAS by ethidium bromide displacement assay

**Figure 3:**
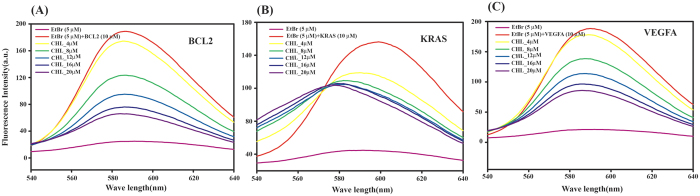
Ethidium bromide (EtBr) displacement assay with increasing concentration of Chelerythrine displaying stacking mode of interaction. (**A**) BCL2 (B) KRAS (**C**) VEGFA. All experiments were carried out using 10 mM potassium phosphate buffer containing 100 mM potassium chloride at pH 7.0.

To explore the mode of binding of Chelerythrine with BCL2, VEGFA and KRAS we performed ethidium bromide displacement assay using fluorescence spectroscopy. Ethidium bromide is known to intercalate with duplex DNA and stacked with G-quadruplex DNA^43^. The fluorescence intensity of ethidium bromide is strongly enhanced upon association with quadruplex sequences as ethidium bromide is stacked and buried by hydrophobic environment of G-quadruplex DNA^44^. Upon addition of Chelerythrine into ethidium bromide-quadruplex complex we observed substantial decrease in fluorescence intensity of ethidium bromide (Fig. 3). This indicates that Chelerythrine stacked with G-face of quadruplex and replaces ethidium bromide.

### Study of secondary conformation of G-quadruplexes in VEGFA, BCL2 and KRAS after complexation with Chelerythrine by circular dichroism Spectroscopy

**Figure 4:**
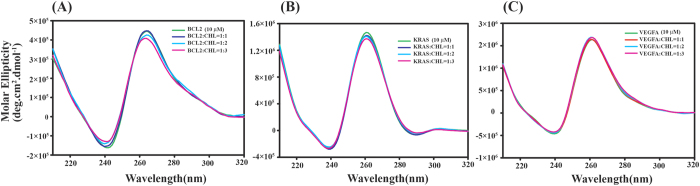
Far UV CD spectra of promoter sequences (BCL2, KRAS, VEGFA) with increasing concentration of Chelerythrine. (**A**) BCL2 (**B**) KRAS (**C**) VEGFA. All experiments were carried out using 10 mM potassium phosphate buffer containing 100 mM potassium chloride at pH 7.0.

CD experiments were performed to examine any conformational changes that may be induced in the quadruplex structure as a result of ligand binding. Parallel G-quadruplex shows positive peak near 260 nm whereas antiparallel G-quadruplex shows a positive peak at 290 nm and a negative peak at 260 nm. Hybrid quadruplex structures show a positive peak at 290 nm, a positive hump at 270 nm and a negative peak at 235 nm^45^. A positive peak near 260 nm for all sequences VEGFA, KRAS and BCL2 clearly indicates formation of parallel G-quadruplex structures (Fig. 4). Upon increasing concentration of Chelerythrine on VEGFA up to a ratio of 1:3 we have seen almost no change in CD spectra of VEGFA. This indicates that Chelerythrine upon binding with VEGFA does not change its overall parallel conformation. For BCL2, we observed a small decrease in molar ellipticity value at 260 nm upon increasing the concentration of Chelerythrine upto a ratio 1:3 but overall parallel topology of BCL2 remains unchanged. A small decrease in molar ellipticity value at 260 nm for KRAS was also observed after complex formation with Chelerythrine without affecting its overall topology. This slight decrease in molar ellipticity may be attributed to association of chromophoric group present in Chelerythrine and quartets of G-quadruplexes^46^.

### Determination of structural stability of Chelerythrine-G-quadruplex complex in VEGFA, BCL2 and KRAS using CD melting experiment

**Figure 5:**
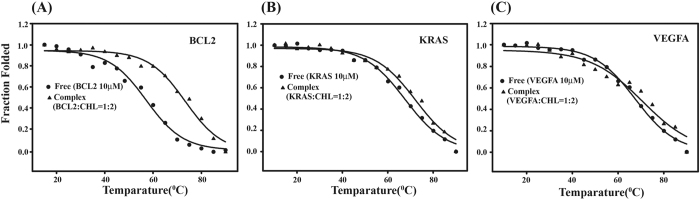
CD melting profile of free promoter sequences (BCL2, KRAS and VEGFA) and their complex with Chelerytrine in the ratio 1:2. (**A**) BCL2 (**B**) KRAS (**C**) VEGFA. All experiments were carried out using 10 mM potassium phosphate buffer containing 100 mM potassium chloride at pH 7.0.

The stability and binding of Chelerythrine-quadruplex complex was investigated by thermal denaturation using CD melting experiment^47^. Thermal melting temperature of free VEGFA, KRAS and BCL2 as well as their complex with Chelerythrine were measured. The melting profiles are reported in Fig. 5. The melting temperature of Chelerythrine-quadruplex complex were increased for BCL2, KRAS and VEGFA compared to that of free quadruplexes (Table S3). Sequences having single nucleotide loop are thermodynamically more stable than sequences having two or more nucleotides in their loops^48^,^49^. BCL2 has only one single nucleotide loop whereas VEGFA and KRAS have two single nucleotide loops in their quadruplex structures thus the melting temperature of free BCL2 (57.1 °C) is lower compared to VEGFA (67.3 °C) and KRAS (67.5 °C). Though the Tm of Chelerythrine-quadruplex complex for VEGFA (70.6 °C), KRAS (72.1 °C) and BCL2 (73.3 °C) are comparable. Increase in melting temperature clearly indicates the complex is thermodynamically more stable than that of the free quadruplex DNA.

### Thermodynamics of binding of Chelerythrine with G-quadruplexes in VEGFA, BCL2 and KRAS by isothermal titration calorimetry

**Figure 6:**
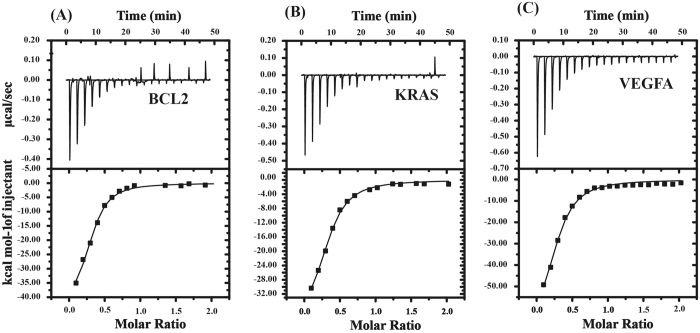
Isothermal titration calorimetry (ITC) profile of Chelerythrine with promoter sequences (BCL2, KRAS and VEGFA). (**A**) BCL2 (**B**) KRAS (**C**) VEGFA. The top panel display the isothermal plot of the Chelerythrine-promoter sequences complex formation, whereas lower panel represent the integrated binding isotherm generated from the integration of peak area as a function of molar ratio. The solid line represents the best fit data using ‘one site binding model’.

The ITC experiment was performed to analyze the energetics of binding between Chelerythrine and promoter sequences. The equilibrium binding constant (K_D_) and changes in thermodynamic parameters such as enthalpy (ΔH), Gibbs free energy (ΔG) and entropy (ΔS) were measured by ITC for all Chelerythrine-quadruplex complexes (BCL2, KRAS, VEGFA)^50^. The ITC profiles for binding of Chelerythrine with promoter sequences are represented in Fig. 6. The thermogram showed single type of binding event for all promoter sequences interacting with Chelerythrine indicating formation of one type of complex. The thermodynamic parameters obtained for the binding of Chelerythrine with quadruplex sequences are given in Table S4. The negative value of enthalpy (∆H) and entropy (∆S) indicates formation of hydrogen bond and van der Waals interaction during binding. The negative value of free energy (∆G) indicates that the binding is spontaneous for Chelerythrine binding to all quadruplex sequences. The binding affinity of Chelerythrine with GC rich duplex DNA was measured by ITC (Figure S3C). Our result furthermore reveals that Chelerythrine has higher binding affinity towards quadruplex of VEGFA, BCL2 and KRAS promoter compared to duplex DNA.

### Chelerythrine treatment downregulates expression of oncogenes in MCF7 cells

**Figure 7:**
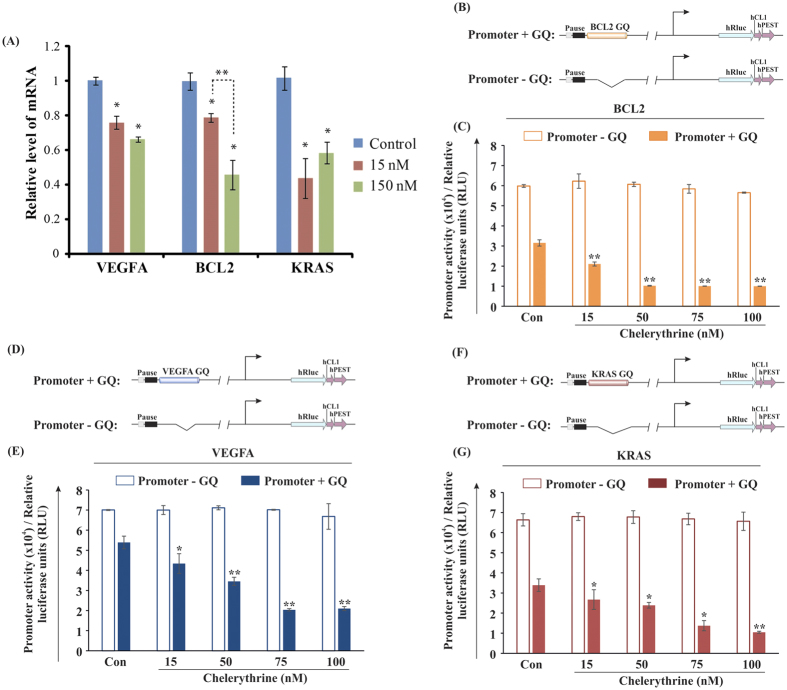
(**A**) Real time PCR analysis show Chelerythrine-mediated repression of VEGFA, KRAS and BCL2 genes expression in MCF7 cells. Bar diagram showing relative expression level, quantified using real time PCR analysis, of VEGFA, KRAS and BCL2 genes after treatment of cells with 15 and 150 nM (n = 3 batches of cells for each treatment) Chelerythrine for 24 h. Control received only the buffer in which Chelerytrine was suspended. Data presented as relative expression level (±Standard Error) where expression in control calculated as almost 1 (±Standard Error). Expressions in different samples normalized based on expression of GAPDH gene. *significantly changed from control, and **significantly changed from cognate 15 nM sample (indicated with dashed line). **(B–G)** Chelerythrine downregulates the promoter activity of BCL2, KRAS and VEGFA through targeting G-quadruplex, confirmed by luciferase assay. (**B,D,F**) Schematic representations of the reporter luciferase constructs are given. BCL2 G-Quadruplex (GQ), KRAS GQ, and VEGFA GQ are the quadruplex- scaffolds which are located at the upstream of *hRlucCP.* GQ deleted constructs are also demonstrated below. (**C,E,G**) Promoter activity is shown in terms of relative luciferase units, which dictates the expression of the reporter gene. Significant reduction in luciferase activity is found for the constructs which harbour the quadruplex motifs in the upstream of promoters compared to those which are devoid of quadruplex motifs. Error bars in the bar plots (**E,F and G**) represent means ± s.d. from three independent experiments. Asterisks (*) indicate statistical significance as determined from Student’s t-test (*indicates P < 0.05, **indicates P < 0.01, ***indicates P < 0.001), which denote significant differences in the promoter activities of BCL2, KRAS, and VEGFA, compared with values for untreated cells (control).

We reasoned that if the ligand Chelerythrine binds to the G-quadruplexes formed in the promoter regions of human VEGFA, KRAS and BCL2 genes, the expression level of them will be negatively affected. We have employed quantitative real time PCR analysis to measure the steady state mRNA level of these three genes after treatment of a human breast cancer cell line, MCF7 where all these three genes were overexpressed, cultured with two different doses of Chelerythrine for 24 h period. Gene specific PCR primers were designed for this purpose and the expression level of GAPDH gene utilized for normalizing data obtained from different samples. For this experiment high quality total RNA (Figure S2A) was used (3 μg), and converted to cDNA using random hexamer primer and a MuMLV based reverse transcriptase. Prior to the real time PCR analysis we have checked the specificity of the designed primers (Figure S2B,C). Amplification of a single major band after 35 cycles of PCR reaction confirmed only gene-specific reactions occurred (Figure S2B). No such amplification was obtained from the -RT control (RT independent) reaction, indicating amplification occurred only from the cDNA (Figure S2C). Next, we performed real time PCR analysis using these cDNAs. Our data show that after 15 nM Chelerythrine treatment expression levels of VEGFA, BCL2 and KRAS were reduced to about 75.7% (p < 0.00004), 78.8% (p < 0.0049) and 42.8% (p < 0.0078) of control, respectively. Increasing the dose to 150 nM did not further change the expression level of VEGFA (66.1% of control, p < 0.000004) and KRAS (57.5% of control, p < 0.0013) genes, indicating 15 nM treatment had saturating effect for these two genes. However, the BCL2 gene showed a significant level of reduced expression after treatment with the higher dose (150 nM) and expression level was reduced to 45.6% of control (p < 0.00017). The molecule did not affect the GAPDH expression in any experiment. These data clearly depict the negative effect of the G-quadruplex binding Chelerythrine on the expression of various chromosomal genes (Fig. 7A).

### Chelerythrine downregulates the promoter activity of BCL2, KRAS, and VEGFA by targeting their upstream quadruplex-forming motifs

We have so far analysed the *in vitro* selectivity and binding affinity of Chelerythrine with the promoter quadruplex elements of BCL2, KRAS, and VEGFA. To advance further, we investigated its intracellular selectivity to these promoter quadruplex scaffolds in MCF-7 cells. We have cloned the promoter sequences of BCL2, KRAS, and VEGFA with or without their corresponding upstream quadruplex elements into a promoter-less pGL4.72 vector, upstream of a luciferase reporter gene (*hRlucCP) (Renilla reniformis*) and transiently transfected them into MCF-7 cells followed by an array of treatment conditions (15–10 nM Chelerythrine) for 24 hours. We reasoned if Chelerythrine could selectively target the promoter quadruplexes inside the cells, the expression of hRlucCP would be downregulated. So, the clones, which are devoid of quadruplex motifs would exhibit higher promoter activity than those having quadruplex scaffolds in the upstream of the *hRlucCP* gene. We have observed significant regression in the luciferase activity in the clones having quadruplex-forming inserts with increasing gradient of Chelerythrine. However, no significant alterations in luciferase activity were found for the constructs without quadruplex-forming motifs (Fig. 7B–G). These findings confirm the efficiency of Chelerythrine for targeting *in cellulo* quadruplexes, but its binding efficiency differs in several magnitudes for these three promoter quadruplex motifs.

### Investigation of atomic level interaction of Chelerythrine-Gquadruplex complex by molecular dynamics simulation

**Figure 8:**
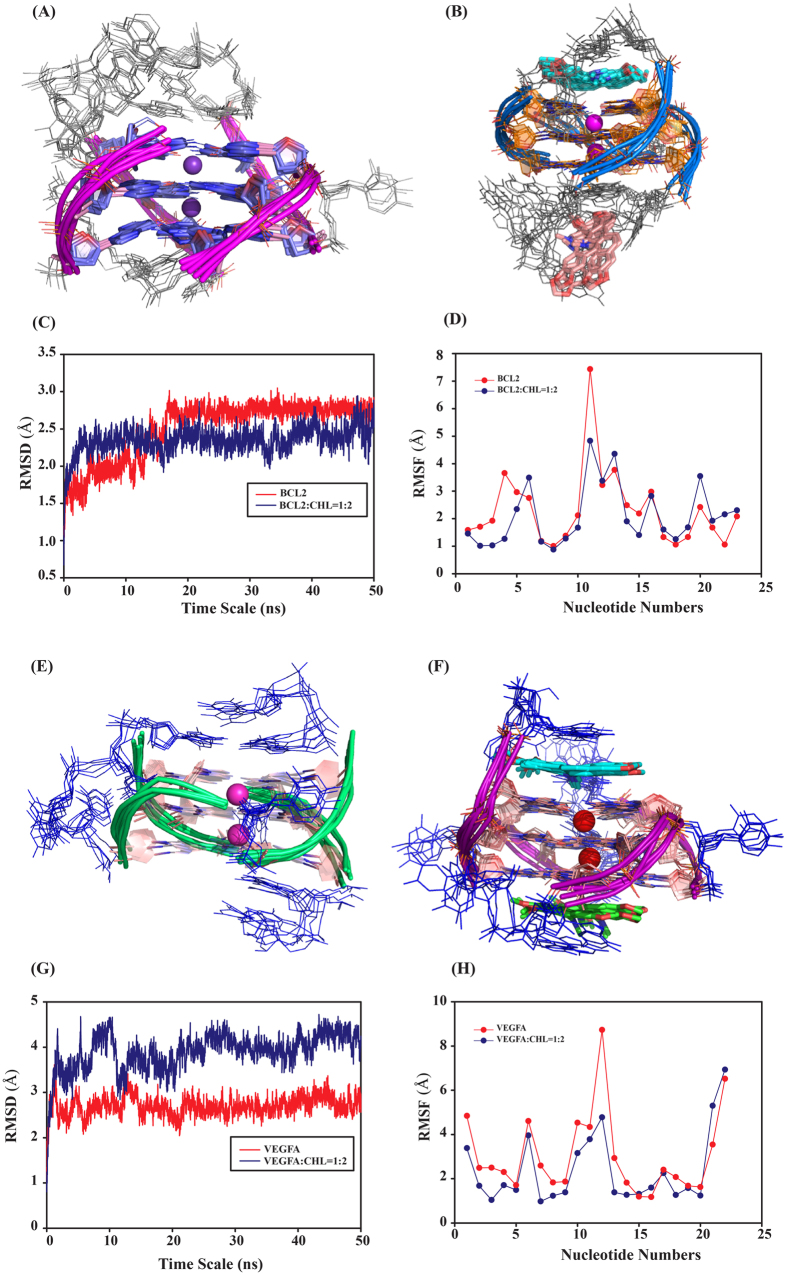
(**A–D**) Ensemble structure of BCL2. (**A**) control (**B**) BCL2-Chelerythrine (1:2) complex. The frames were collected from 45–50 ns of MD trajectory. (grey colour showed DNA bases present in the loop) (blue (**A**) and orange (**B**) color showed G-quartet forming bases) (**C**) A plot of RMSD of all atom vs time. (**D**) A plot of RMSF of individual residue of BCL2 control and BCL2-Chelerythrine complex. (**E–H**) Ensemble structure of VEGFA. (**E**) control (**F**) VEGFA-Chelerythrine (1:2) complex. The frames were collected from 45-50 ns of MD trajectory. (blue colour showed DNA bases present in the loop). (light pink (**A**) and (**B**) color showed G-quartet forming bases) (**G**) A plot of RMSD of all atom vs time. (**H**) A plot of RMSF of individual residue of BCL2 control and VEGFA-Chelerythrine complex.

Molecular dynamics simulation showed insights of mechanism of binding of Chelerythrine-quadruplex interaction. MD simulation gives the detailed information of complex formation at the atomic level^51^,^52^. In explicit solvent medium Chelerythrine adjust its position with respect to initial docked state^53^. The binding ratio obtained from ITC was found to be 1:2 for quadruplex-Chelerythrine complex for all promoter sequences. Hence we docked Chelerythrine and quadruplex upto 1:2 ratio. First, Chelerythrine was docked at the 3′-end face (**G3-G7-G19-G23**) of BCL2 (PDB ID: 2F8U)^18^ to obtain 1:1 complex (Figure S4A). Mainly it stacked with **G3** & **G7** nucleotides. The second round of docking were performed using 1:1 complex to obtain 1:2 complex. The second Chelerythrine was found to stack with loop bases (**G11-A14-T15**) (Figure S4B). To gain more information at atomic label MD simulation was performed for 50 ns for BCL2 control and BCL2-Chelerythrine 1:2 complex. The average structures of BCL2 control obtained from MD simulation were superimposed from the final 10 ns of the trajectory at an interval of 2 ns (Fig. 8A). Similarly the average structures of BCL2-Chelerythrine complex were superimposed at the final 10 ns of MD simulation (Fig. 8B). Figure 8C showed the RMSD plot of BCL2 control and BCL2-Chelerytrine 1:2 complex over the simulation time scale. An initial jump in the RMSD scale was found for first 5 ns for BCL2 control and 10 ns for Complex compared to that of the starting frame due to relaxation of the model. The stability of the trajectories of the complex can be observed with minimal fluctuation ranging from 1.5 to 2.8 A°. The RMSD plot of complex was found to be stable over the entire simulation time scale which concludes that Chelerythrine stabilizes BCL2. Figure 8D showed Root Mean Square Fluctuation (RMSF) of the nucleotides for BCL2 control and BCL2-Chelerythrine complex. Interestingly we have seen that fluctuation in loop nucleotides are more compared to that in the G-nucleotides forming core G-quartets. We observed G-face as well as groove binding of Chelerythrine to G-quadruplex over the simulation trajectory. In the conformational hyperspace of Chelerythrine binding to G-quadruplex (BCL2) it is reflected that G-face binding of the ligand is energetically more favored compared that of the groove binding as evidenced from the MMPBSA calculation (Table S7, ΔG_G-face_ = −24.1 kcal/mole, ΔG_Groove_ = −13.9 kcal/mole). For VEGFA (PDB ID: 2M27)^54^, we found that first Chelerythrine was docked at 5′-end face (**G3-G7-G14-G18**) (Figure S5A) and then second Chelerythrine docked at the 3′-end face (**G5-G9-G16-G20**) of VEGFA (Figure S5B). Similarly, 50 ns of MD simulation were performed with VEGFA*-*Chelerythrine complex and VEGFA control. The superimposed structures collected from final 10 ns of MD simulation snapshots were found to be very stable for VEGFA control (Fig. 8E) and VEGFA-Chelerythrine complex (Fig. 8F). Figure 8G showed RMSD plot of VEGFA control and VEGFA-Chelerythrine complex throughout the simulation timescale. RMSD of complex found to be stable after 10 ns which indicate Chelerythrine form stable complex with VEGFA. A plot of RMSF vs. nucleotides (Residue) (Fig. 8H) showed that loop nucleotides were more flexible compared to nucleotides in the G-faces. Chelerythrine stacks on 3′ and 5′- end faces with a small energy difference reflected from MMPBSA data (Table S8, ΔG_G-face(5′)_ = −22.1 kcal/mole, ΔG _G-face(3′)_ = −17.6 kcal/mole).

The thermodynamic parameters calculated using MMPBSA method, in this context the theoretical results are valuable to be compared with the experimental findings^53^. The binding energies of quadruplex-Chelerythrine (1:1 and 1:2) complex are shown in Tables S7 and S8.

## Discussion

Earlier reports suggested that anticancer activity of Chelerythrine, a naturally occurring benzophenanthridine plant alkaloid is associated with its potential ability to inhibit protein kinese C. It is also reported previously that Chelerythrine is recognized by human telomeric DNA and RNA G-quadruplex^34^,^53^. In the present study we have shown that Chelerythrine not only binds to human telomeric DNA and RNA G-quadruplex it also binds to quadruplex structures formed in the promoter region of oncogenes like BCL2, VEGFA and KRAS. Various spectroscopic techniques (UV absorption spectroscopy, fluorescence anisotropy, circular dichroism spectroscopy and CD melting) and molecular dynamics simulation fruitfully monitored the binding of Chelerythrine to these G quadruplex structures. Isothermal titration calorimetry in conjunction with MMPBSA calculation confirmed that the Chelerythrine-quadruplex complex formation is thermodynamically feasible process. RT-PCR experiment validated that Chelerythrine can efficiently arrest promoter G-quadruplex structures resulting the down regulation of the gene expression. This study enables us to validate the depth of existence of G quadruplexes modulating the transcriptional regulations of BCL2, KRAS and VEGFA.

The observed red shift (12–14 nm) and hypochromism (50–63%) in the absorption spectra of Chelerythrine with increasing concentration of promoter sequences indicate strong complex formation. The π* orbital of Chelerythrine is stabilized by the transition of π electron from DNA bases, thereby reducing the energy gap between π (Chelyrethrine)-π*(DNA) orbital resulting in red shift in the absorption spectra. The hypochromism and red shift indicate end stacking mode of binding for all Chelerythrine-quadruplex complexes. In addition, there is an increase in fluorescence anisotropy of Chelerythrine upon binding with promoter sequences. Increase in fluorescence anisotropy is due to restricted motion of Chelerythrine upon formation of complex with quadruplex compared to that of in the free Chelerythrine. The dissociation constants (K_D_) obtained from fluorescence anisotropy and absorption spectroscopy were almost comparable and are in good agreement with previously reported literature on plant alkaloids and others small molecules binding to G-quadruplex structures^55^. Ethidium bromide is known to bind with G-quadruplex DNA by end stacking mode^43^. Successive addition of Chelerythrine on ethidium bromide-quadruplex complex showed a decrease in fluorescence intensity of ethidium bromide. This indicates that Chelerythrine displaces ethidium bromide and binds to quadruplex sequences by end stacking mode. This result has been well correlated with that of UV absorption and docking studies. From molecular docking we have seen that Chelerythrine stacked with 5′-and 3′-end faces with VEGFA. It has been previously reported that planar aromatic moiety interacts with G-quadruplex through end stacking to the plane of quartet on one or both ends of G-quadruplex DNA^46^,^56^. In case of BCL2, one Chelerythrine stacked at the 3′-end face and second one binds with loop bases (G11-A14-T15) of BCL2. This type of interaction mode (stacking on G-face as well on loop bases) was previously reported for sanguinarine binding to Pu27 by Ghosh *et al*. where two molecules stack at two ends and 3rd one at loop bases^46^. The observed CD spectra showed that all the promoter sequences formed parallel G-quadruplex structrure in presence of K^+^ ions. There is no notable change in the CD spectra of all the promoter sequences after binding to Chelerythrine. The increased melting temperatures for Chelerythrine-quadruplex complexes in comparison to that of the free quadruplexes indicate thermodynamic stabilization of the complexes. The negative enthalpic contribution obtained from ITC reveals stacking mode of interaction between Chelerythrine and promoter sequences. The negative value of ∆G indicates the interactions of Chelerythrine with all three quadruplexes are spontaneous in nature. The binding constant (K_D_) obtained from ITC is closed to other spectroscopic data. The binding affinity (K_A_) of Chelerythrine-quadruplex complexes with promoter sequences (BCL2, VEGF and KRAS) is significantly higher than Chelerythrine binding with human telomeric G-quadruplex^53^. Interestingly the binding affinities are in good agreement with other potent G-quadruplex binders serving as promising anticancer drug targets^57^. ITC results could be well correlated with docking and MMPBSA data. The details of mechanism of binding at atomic level were further correlated using MD Simulation studies. The presence of conjugated aromatic system in ligand can efficiently stacked on quartet^58^. Chelerythrine has four conjugated aromatic ring which can efficiently stack on G-face of quartet or with the loop bases by π-π stacking interaction. Molecular docking of Chelerythrine with BCL2 demonstrated that one Chelerythrine stacked with 3′-end face (**G3-G7-G19-G23**) and second one interact with loop bases (**G11-A14-T15**) whereas in case of VEGFA two Chelerythrine docked at 5′ end face (**G3-G7-G14-G18**) and 3′ end face (**G5-G9-G16-G20**) by end stacking mode. Our docking results strengthen the results obtained from spectroscopic and calorimetric data. The ensemble structures obtained from the last 5 ns simulation time with an interval of 1 ns of Chelerythrine-quadruplex complex (BCL2 and VEGFA) indicate stable complex formation with minimal backbone rmsd. The stability of Chelerythrine-quadruplex complexes were also reflected in their RMSD profiles. From the RMSD and RMSF profile it may be concluded that the G-quadruplex were stable (both in control and complex) over the entire course of simulation time. The binding free energy thermodynamic parameters obtained from MMPBSA calculation showed that 3′ face binding affinity of Chelerythrine is energetically more favored than interaction with loop bases with BCL2.

A small molecule capable of arresting the G-quadruplex structure that is located in the promoter region of an oncogene, brings in transcriptional repression of the gene^6^,^10^. This is a highly suggested therapeutic approach for treatment of different carcinomas in which these oncogenes are overexpressed^10^. Among many oncogenes, VEGFA, BCL2 and KRAS are overexpressed in many types of cancer cells. Our data (RT-PCR and luciferase assay) strongly suggest that treatment of Chelerythrine on MCF7 cells downregulate transcript levels of these three genes significantly within 24 hours at nanomolar level. During this treatment cells also remained unaffected in terms of their morphology. Our biophysical experiments confirmed that Chelerythrine strongly binds to the G-quadruplexes in promoters of these three genes. Taken together the biophysical results and RT-PCR data, it could be inferred that downregulation of VEGFA, BCL2 and KRAS expression in MCF7 cells after Chelerythrine treatment was due to interaction of the molecule with the G-quadruplexes of their promoters. The slight increase in KRAS expression in 150 nM treated samples compared to the expression in 15 nM Chelerythrine treated samples was not significant, and may have arisen due to experimental variation, however, both treatments resulted in significant downregulation of KRAS expression compared to the control (Fig. 7A). Reduction of luciferase activity having quadruplex forming inserts with increasing concentration of Chelerythrine confirms that Chelerythrine efficiently binds to in *cellulo* quadruplex motifs of these three genes (VEGFA, BCL2 and KRAS) (Fig. 7E–G). Chelerythrine has been established as a potent and specific inhibitor of protein kinase C with an IC_50_ (concentration causing a 50% inhibition) value of 0.66 μM^31^. Chelerythrine exerts its anticancer activity by stabilizing G-quadruplex structure as well as by inhibition of protein kinase C. The RT-PCR experiment in combination with luciferase assay showed that Chelerythrine treatment to MCF7 breast cancer cells highly down regulated the transcript level of VEGFA, BCL2 and KRAS genes, suggesting that arresting of promoter G-quadruplex structures resulting the repression of transcription of these genes at nanomolar level. We can conclude that G-quadruplex binding property of Chelerythrine plays a very crucial role for the anticancer property of the compound.

The BCL2 gene plays an essential role in cell survival acting as an inhibitor of cell apoptosis and connected with anti-apoptotic proteins. Vascular endothelial growth factor (VEGFA) is angiogenic growth factor that plays a crucial role in angiogenesis and tumor progression. KRAS which is one of the most frequently mutated oncogenes has a role in many signal transduction pathways significant to different types of human carcinomas including colorectal, pancreatic and lung. These genes are overexpressed in many types of human cancers. The various spectroscopic, thermodynamic, MD simulation techniques, RT-PCR data and luciferase assay establishes the fact that Chelerythrine binds to G-quadruplex structures at the promoter of these genes which leads to down regulate their expression.

## Additional Information

**How to cite this article**: Jana, J. *et al*. Chelerythrine down regulates expression of VEGFA, BCL2 and KRAS by arresting G-Quadruplex structures at their promoter regions. *Sci. Rep.* **7**, 40706; doi: 10.1038/srep40706 (2017).

**Publisher's note:** Springer Nature remains neutral with regard to jurisdictional claims in published maps and institutional affiliations.

## Supplementary Material

Supplementary Information

## References

[b1] Watson J. D. & Crick F. H. Molecular structure of nucleic acids; a structure for deoxyribose nucleic acid. Nature 171, 737–738 (1953). 13054692 10.1038/171737a0

[b2] Brooks T. A. & Hurley L. H. The role of supercoiling in transcriptional control of MYC and its importance in molecular therapeutics. Nat Rev Cancer 9, 849–861 (2009). 19907434 10.1038/nrc2733

[b3] Siddiqui-Jain A., Grand C. L., Bearss D. J. & Hurley L. H. Direct evidence for a G-quadruplex in a promoter region and its targeting with a small molecule to repress c-MYC transcription. Proc Natl Acad Sci USA 99, 11593–11598 (2002). 12195017 10.1073/pnas.182256799 PMC129314

[b4] Bochman M. L., Paeschke K. & Zakian V. A. DNA secondary structures: stability and function of G-quadruplex structures. Nat Rev Genet 13, 770–780 (2012). 23032257 10.1038/nrg3296 PMC3725559

[b5] Verma A., Yadav V. K., Basundra R., Kumar A. & Chowdhury S. Evidence of genome-wide G4 DNA-mediated gene expression in human cancer cells. Nucleic Acids Res 37, 4194–4204 (2009). 19211664 10.1093/nar/gkn1076 PMC2715224

[b6] Balasubramanian S., Hurley L. H. & Neidle S. Targeting G-quadruplexes in gene promoters: a novel anticancer strategy? Nat Rev Drug Discov 10, 261–275 (2011). 21455236 10.1038/nrd3428 PMC3119469

[b7] Hanahan D. & Weinberg R. A. Hallmarks of cancer: the next generation. Cell 144, 646–674 (2011). 21376230 10.1016/j.cell.2011.02.013

[b8] Phan A. T., Kuryavyi V., Gaw H. Y. & Patel D. J. Small-molecule interaction with a five-guanine-tract G-quadruplex structure from the human MYC promoter. Nat Chem Biol 1, 167–173 (2005). 16408022 10.1038/nchembio723 PMC4690526

[b9] Cui X., Lin S. & Yuan G. Spectroscopic probing of recognition of the G-quadruplex in c-kit promoter by small-molecule natural products. Int J Biol Macromol 50, 996–1001 (2012). 22405847 10.1016/j.ijbiomac.2012.02.029

[b10] Lavrado J. . KRAS oncogene repression in colon cancer cell lines by G-quadruplex binding indolo[3,2-c]quinolines. Sci Rep 5, 9696 (2015). 25853628 10.1038/srep09696 PMC5382548

[b11] Sheu S. Y., Huang C. H., Zhou J. K. & Yang D. Y. Relative stability of G-quadruplex structures: Interactions between the human Bcl2 promoter region and derivatives of carbazole and diphenylamine. Biopolymers 101, 1038–1050 (2014). 24723333 10.1002/bip.22497

[b12] Wu Y. . Stabilization of VEGF G-quadruplex and inhibition of angiogenesis by quindoline derivatives. Biochim Biophys Acta 1840, 2970–2977 (2014). 24931695 10.1016/j.bbagen.2014.06.002

[b13] Kyo S., Takakura M., Fujiwara T. & Inoue M. Understanding and exploiting hTERT promoter regulation for diagnosis and treatment of human cancers. Cancer Sci 99, 1528–1538 (2008). 18754863 10.1111/j.1349-7006.2008.00878.x PMC11158053

[b14] Rankin S. . Putative DNA quadruplex formation within the human c-kit oncogene. J Am Chem Soc 127, 10584–10589 (2005). 16045346 10.1021/ja050823u PMC2195896

[b15] Cogoi S. & Xodo L. E. G-quadruplex formation within the promoter of the KRAS proto-oncogene and its effect on transcription. Nucleic Acids Res 34, 2536–2549 (2006). 16687659 10.1093/nar/gkl286 PMC1459413

[b16] Dai J. . An intramolecular G-quadruplex structure with mixed parallel/antiparallel G-strands formed in the human BCL-2 promoter region in solution. J Am Chem Soc 128, 1096–1098 (2006). 16433524 10.1021/ja055636a PMC2556172

[b17] Sun D., Guo K., Rusche J. J. & Hurley L. H. Facilitation of a structural transition in the polypurine/polypyrimidine tract within the proximal promoter region of the human VEGF gene by the presence of potassium and G-quadruplex-interactive agents. Nucleic Acids Res 33, 6070–6080 (2005). 16239639 10.1093/nar/gki917 PMC1266068

[b18] Dai J., Chen D., Jones R. A., Hurley L. H. & Yang D. NMR solution structure of the major G-quadruplex structure formed in the human BCL2 promoter region. Nucleic Acids Res 34, 5133–5144 (2006). 16998187 10.1093/nar/gkl610 PMC1636422

[b19] Catz S. D. & Johnson J. L. BCL-2 in prostate cancer: a minireview. Apoptosis 8, 29–37 (2003). 12510149 10.1023/a:1021692801278

[b20] Binder C. . Bcl-2 protein expression in breast cancer in relation to established prognostic factors and other clinicopathological variables. Ann Oncol 6, 1005–1010 (1995). 8750153 10.1093/oxfordjournals.annonc.a059064

[b21] Sinicrope F. A. . bcl-2 and p53 oncoprotein expression during colorectal tumorigenesis. Cancer Res 55, 237–241 (1995). 7812951

[b22] McMahon G. VEGF receptor signaling in tumor angiogenesis. Oncologist 5 Suppl 1, 3–10 (2000). 10804084 10.1634/theoncologist.5-suppl_1-3

[b23] Prenen H., Tejpar S. & Van Cutsem E. New strategies for treatment of KRAS mutant metastatic colorectal cancer. Clin Cancer Res 16, 2921–2926 (2010). 20460490 10.1158/1078-0432.CCR-09-2029

[b24] di Magliano M. P. & Logsdon C. D. Roles for KRAS in pancreatic tumor development and progression. Gastroenterology 144, 1220–1229 (2013). 23622131 10.1053/j.gastro.2013.01.071 PMC3902845

[b25] Riely G. J., Marks J. & Pao W. KRAS mutations in non-small cell lung cancer. Proc Am Thorac Soc 6, 201–205 (2009). 19349489 10.1513/pats.200809-107LC

[b26] Ohnmacht S. A. & Neidle S. Small-molecule quadruplex-targeted drug discovery. Bioorg Med Chem Lett 24, 2602–2612 (2014). 24814531 10.1016/j.bmcl.2014.04.029

[b27] Duan W. . Design and synthesis of fluoroquinophenoxazines that interact with human telomeric G-quadruplexes and their biological effects. Mol Cancer Ther 1, 103–120 (2001). 12467228

[b28] Chung W. J., Heddi B., Hamon F., Teulade-Fichou M. P. & Phan A. T. Solution structure of a G-quadruplex bound to the bisquinolinium compound Phen-DC(3). Angew Chem Int Ed Engl 53, 999–1002 (2014). 24356977 10.1002/anie.201308063

[b29] Zhang S., Wu Y. & Zhang W. G-quadruplex structures and their interaction diversity with ligands. ChemMedChem 9, 899–911 (2014). 24729465 10.1002/cmdc.201300566

[b30] Murat P., Singh Y. & Defrancq E. Methods for investigating G-quadruplex DNA/ligand interactions. Chem Soc Rev 40, 5293–5307 (2011). 21720638 10.1039/c1cs15117g

[b31] Herbert J. M., Augereau J. M., Gleye J. & Maffrand J. P. Chelerythrine is a potent and specific inhibitor of protein kinase C. Biochem Biophys Res Commun 172, 993–999 (1990). 2244923 10.1016/0006-291x(90)91544-3

[b32] Chen X. M., Zhang M., Fan P. L., Qin Y. H. & Zhao H. W. Chelerythrine chloride induces apoptosis in renal cancer HEK-293 and SW-839 cell lines. Oncol Lett 11, 3917–3924 (2016). 27313717 10.3892/ol.2016.4520 PMC4888265

[b33] Chmura S. J. . *In vitro* and *in vivo* activity of protein kinase C inhibitor chelerythrine chloride induces tumor cell toxicity and growth delay *in vivo*. Clin Cancer Res 6, 737–742 (2000). 10690561

[b34] Bai L. P., Hagihara M., Nakatani K. & Jiang Z. H. Recognition of chelerythrine to human telomeric DNA and RNA G-quadruplexes. Sci Rep 4, 6767 (2014). 25341562 10.1038/srep06767 PMC4208030

[b35] Ghosh A. . Sequence context induced antimicrobial activity: insight into lipopolysaccharide permeabilization. Mol Biosyst 10, 1596–1612 (2014). 24714742 10.1039/c4mb00111g

[b36] Schmittgen T. D. & Livak K. J. Analyzing real-time PCR data by the comparative C(T) method. Nature protocols 3, 1101–1108 (2008). 18546601 10.1038/nprot.2008.73

[b37] Wang J., Wang W., Kollman P. A. & Case D. A. Automatic atom type and bond type perception in molecular mechanical calculations. J Mol Graph Model 25, 247–260 (2006). 16458552 10.1016/j.jmgm.2005.12.005

[b38] Dang L. X. Mechanism and thermodynamics of ion selectivity in Aquous Solutions of 18-Crown-6 Ether: A Molecular Dynamics Study. Journal of American Chemical Society 117, 7 (1995).

[b39] Shan Y., Klepeis J. L., Eastwood M. P., Dror R. O. & Shaw D. E. Gaussian split Ewald: A fast Ewald mesh method for molecular simulation. J Chem Phys 122, 54101 (2005). 15740304 10.1063/1.1839571

[b40] Krutler V., Gunsteren W. F. V. & Hunenberger P. H. A fast SHAKE algorithm to solve distance constraint equations for small molecules in molecular dynamics simulations. Journal of Computational Chemistry 22, 501–508 (2001).

[b41] Roe D. & Cheatham T. E. PTRAJ and CPPTRAJ: Software for Processing and Analysis of Molecular Dynamics Trajectory Data. Journal of Chemical Theory and Computation 9, 3084–3095 (2013). 26583988 10.1021/ct400341p

[b42] Wei C., Jia G., Yuan J., Feng Z. & Li C. A spectroscopic study on the interactions of porphyrin with G-quadruplex DNAs. Biochemistry 45, 6681–6691 (2006). 16716079 10.1021/bi052356z

[b43] Guo Q., Lu M., Marky L. A. & Kallenbach N. R. Interaction of the dye ethidium bromide with DNA containing guanine repeats. Biochemistry 31, 2451–2455 (1992). 1547228 10.1021/bi00124a002

[b44] Koeppel F. . Ethidium derivatives bind to G-quartets, inhibit telomerase and act as fluorescent probes for quadruplexes. Nucleic Acids Res 29, 1087–1096 (2001). 11222758 10.1093/nar/29.5.1087 PMC29720

[b45] Paramasivan S., Rujan I. & Bolton P. H. Circular dichroism of quadruplex DNAs: applications to structure, cation effects and ligand binding. Methods 43, 324–331 (2007). 17967702 10.1016/j.ymeth.2007.02.009

[b46] Ghosh S., Pradhan S. K., Kar A., Chowdhury S. & Dasgupta D. Molecular basis of recognition of quadruplexes human telomere and c-myc promoter by the putative anticancer agent sanguinarine. Biochim Biophys Acta 1830, 4189–4201 (2013). 23562763 10.1016/j.bbagen.2013.03.027

[b47] Lane A. N., Chaires J. B., Gray R. D. & Trent J. O. Stability and kinetics of G-quadruplex structures. Nucleic Acids Res 36, 5482–5515 (2008). 18718931 10.1093/nar/gkn517 PMC2553573

[b48] Guédin A., Gros J., Alberti P. & Mergny J. L. How long is too long? Effects of loop size on G-quadruplex stability. Nucleic Acids Res 38, 7858–7868 (2010). 20660477 10.1093/nar/gkq639 PMC2995061

[b49] Guo K., Gokhale V., Hurley L. H. & Sun D. Intramolecularly folded G-quadruplex and i-motif structures in the proximal promoter of the vascular endothelial growth factor gene. Nucleic Acids Res 36, 4598–4608 (2008). 18614607 10.1093/nar/gkn380 PMC2504309

[b50] Di Leva F. S., Novellino E., Cavalli A., Parrinello M. & Limongelli V. Mechanistic insight into ligand binding to G-quadruplex DNA. Nucleic Acids Res 42, 5447–5455 (2014). 24753420 10.1093/nar/gku247 PMC4027208

[b51] Jana J. . Human cathelicidin peptide LL37 binds telomeric G-quadruplex. Mol Biosyst 9, 1833–1836 (2013). 23636272 10.1039/c3mb70030e

[b52] Banerjee V. . Use of a small peptide fragment as an inhibitor of insulin fibrillation process: a study by high and low resolution spectroscopy. PLoS One 8, e72318 (2013). 24009675 10.1371/journal.pone.0072318 PMC3756998

[b53] Ghosh S., Jana J., Kar R. K., Chatterjee S. & Dasgupta D. Plant alkaloid chelerythrine induced aggregation of human telomere sequence–a unique mode of association between a small molecule and a quadruplex. Biochemistry 54, 974–986 (2015). 25566806 10.1021/bi501117x

[b54] Agrawal P., Hatzakis E., Guo K., Carver M. & Yang D. Solution structure of the major G-quadruplex formed in the human VEGF promoter in K+: insights into loop interactions of the parallel G-quadruplexes. Nucleic Acids Res 41, 10584–10592 (2013). 24005038 10.1093/nar/gkt784 PMC3905851

[b55] Arora A. . Binding of berberine to human telomeric quadruplex - spectroscopic, calorimetric and molecular modeling studies. FEBS J 275, 3971–3983 (2008). 18616467 10.1111/j.1742-4658.2008.06541.x

[b56] Barbieri C. M. . Defining the mode, energetics and specificity with which a macrocyclic hexaoxazole binds to human telomeric G-quadruplex DNA. Nucleic Acids Res 35, 3272–3286 (2007). 17452355 10.1093/nar/gkm188 PMC1904271

[b57] Pagano B., Mattia C. A. & Giancola C. Applications of isothermal titration calorimetry in biophysical studies of G-quadruplexes. Int J Mol Sci 10, 2935–2957 (2009). 19742177 10.3390/ijms10072935 PMC2738904

[b58] Dhamodharan V., Harikrishna S., Jagadeeswaran C., Halder K. & Pradeepkumar P. I. Selective G-quadruplex DNA stabilizing agents based on bisquinolinium and bispyridinium derivatives of 1,8-naphthyridine. J Org Chem 77, 229–242 (2012). 22126189 10.1021/jo201816g

