# Supplementary material for: Chelerythrine down regulates expression of VEGFA, BCL2 and KRAS by arresting G-Quadruplex structures at their promoter regions
Source: Sci Rep. 2017 Jan 19;7:40706. doi: 10.1038/srep40706 (PMC5244364; doi:10.1038/srep40706)
Supplement: Supplementary Information [file srep40706-s1.pdf]

Chelerythrine down regulates expression of VEGFA, BCL2 and KRAS by arresting G-Quadruplex structures at their promoter regions

Jagannath Jana[1], Soma Mondal[1], Payel Bhattacharjee[2], Pallabi Sengupta[1], Tanaya Roychowdhury[3], Pranay Saha[1], Pallob Kundu[2] and Subhrangsu Chatterjee[1]*

[1]Department of Biophysics, Bose Institute, Kolkata, WB, India
[2]Division of Plant Biology, Bose Institute, Kolkata ,WB, India
[3] Cancer Biology and Inflammatory Disorder Division, Kolkata, WB, India

E-mail: subhro_c@jcbose.ac.in

Methods:

Binding energy calculation method:

MD simulation trajectories were analysed with MMPBSA method to evaluate binding energy. The energies and binding energy of individual ligand, receptor and complex were calculated using the GP/PB methods as per Equation S1 and Equation S2 respectively.

Binding energy (G_{Total}) = $E_{mm} + G_{solvation}$ Equation S1

$E_{mm} = E_{vdw} + E_{elec}$ Equation S2

Gsolvation was determined using electrostatic contribution of solvation energy of the PB approach (ΔG_{PB}) and non-polar contribution of solvation energy (ΔGnp).

Entropy contribution TΔS was calculated using the nmode analysis. Binding energy of Chelerythrine and quadruplex was calculated using the Equation S3.

$\Delta G_{Bind} = Gtotal - T\Delta S$ Equation S3

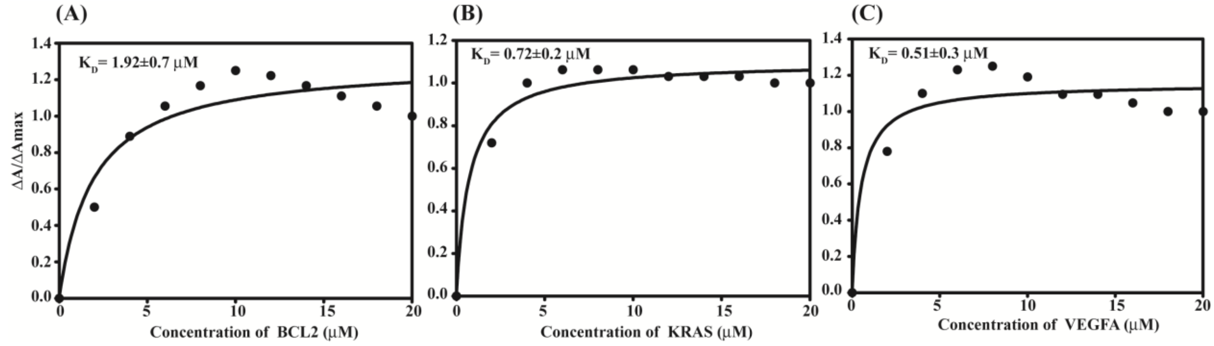

Figure S1: A plot of $\Delta A/\Delta A_{max,316}$ vs. promoter concentration gives us dissociation constant.

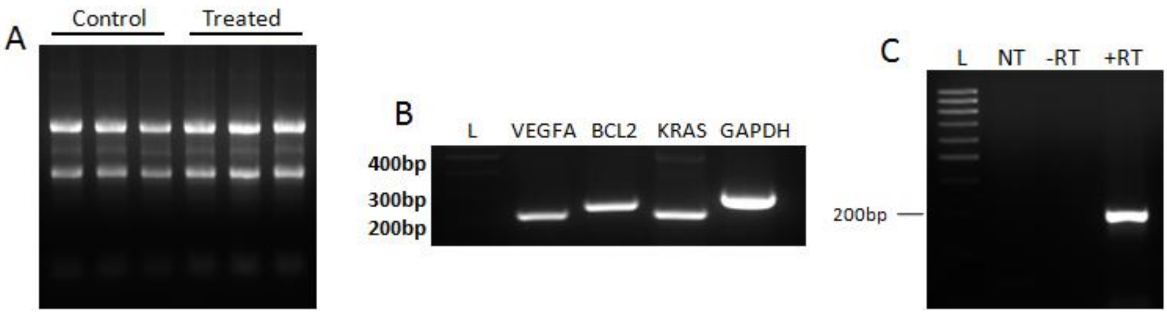

Figure S2: Quantification of expression level of three cellular genes in MCF7 cells treated with Chelerythrine. Agarose gel photographs of denatured total RNA from control (buffer treated) and Chelerythrine treated cells (A); B, end products of PCR reaction carried out using primers for VEGFA, KRAS, BCL2 and GAPDH genes; and C, GAPDH PCR end products along with control reactions. Amplification of a single major band obtained in most of the PCR reactions, confirming specificity of the designed primers. L, DNA ladder, NT, no template (GAPDH PCR reaction without a valid template) control and –RT, RT independent PCR reaction with GAPDH primer pair.

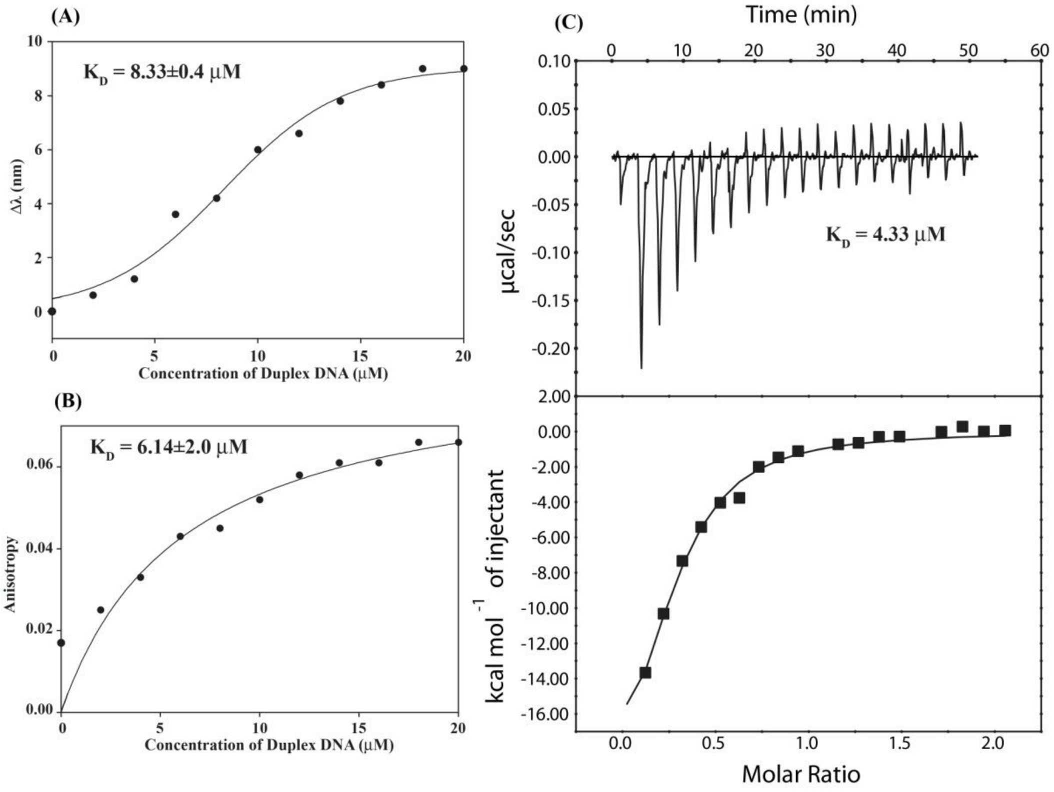

Figure S3: (A) A plot of Δλ vs. concentration of duplex DNA gives us dissociation constant(UV absorption spectroscopy). (B) Fluorescence Anisotropy of Chelerythrine (10 μM) vs incresaing concentration of duplex DNA. (C) Isothermal titration calorimetry (ITC) profile of Chelerythrine with duplex DNA. All experiments were carried out using 10 mM potassium phosphate buffer containing 100 mM potassium chloride at pH 7.0.

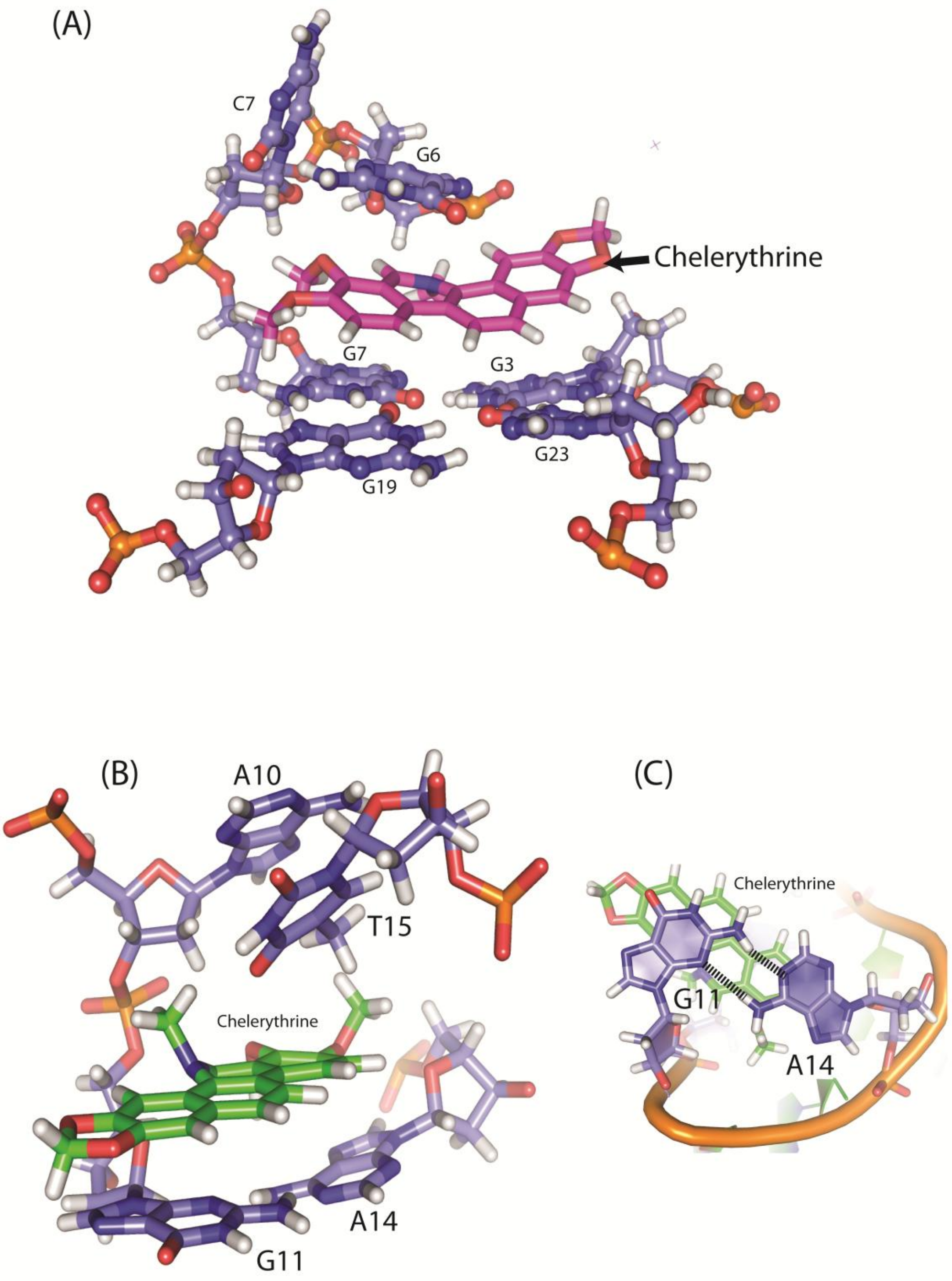

Figure S4: (A) One Chelerythrine (stick) stack with 3'-end face of BCL2 (ball and stick). (B) Second Chelerythrine (stick) stack with loop bases of BCL2 (stick). (C) Base pair formation between G11 and A14 of BCL2 and stacking with Chelerythrine.

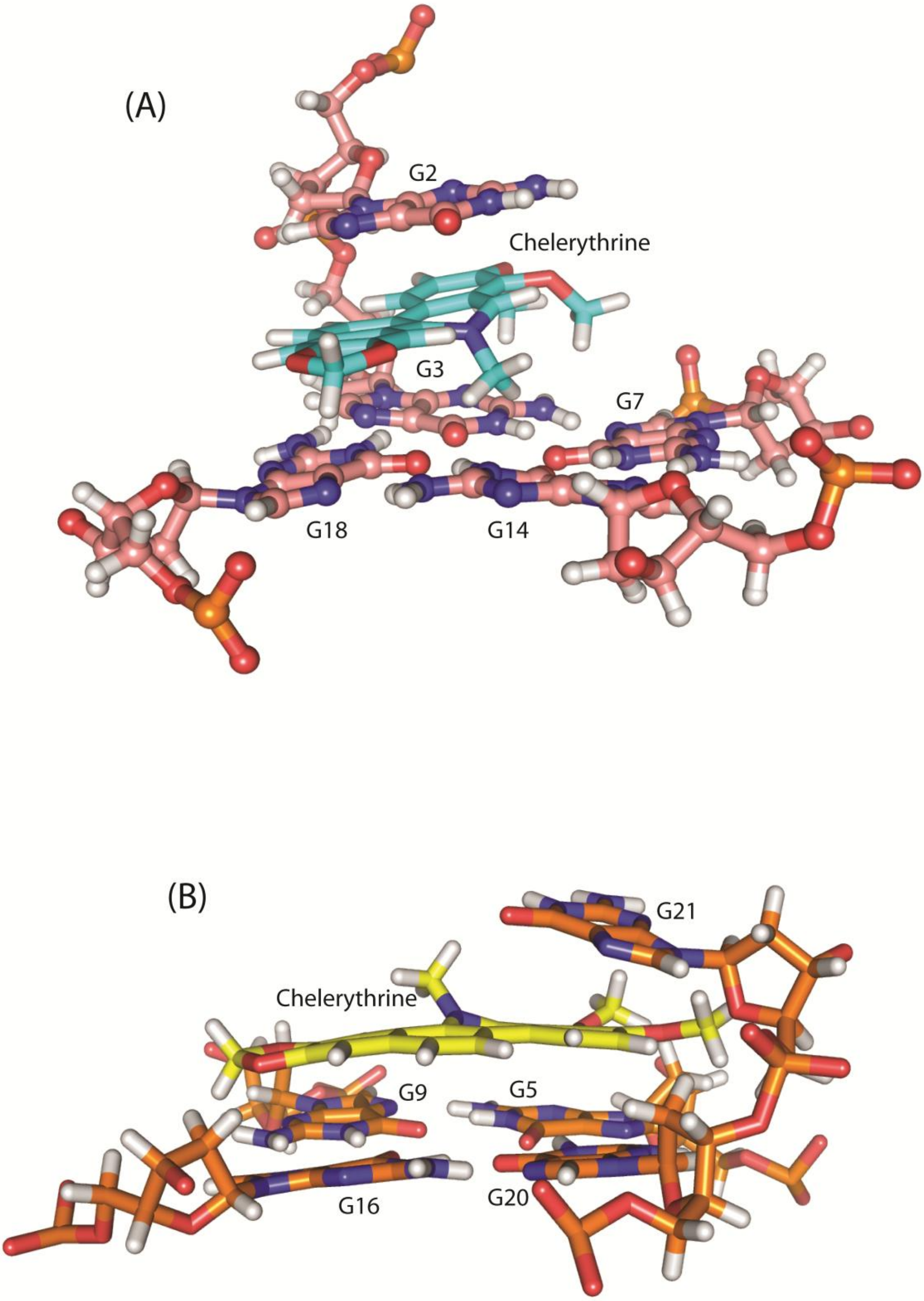

Figure S5: (A) One Chelerythrine (stick) with 5'- end face of VEGFA (ball and stick). (B) Second Chelerythrine stack with 3'- end face of VEGFA.

Table S1: Red shift and dissociation constant obtained from UV spectroscopy.

Parameters	BCL2	KRAS	VEGFA
**Red Shift(nm)**	12	14	12
**K_D(μM)**	1.92±0.7	0.72±0.2	0.51±0.3
**Hypochromism**	61%	50%	63%

Table S2: Dissociation constant obtained from Fluorescence Anisotropy.

Sequences	BCL2	KRAS	VEGFA
**K_D(μM)**	3.2	1.5	0.25

Table S3: Melting temperature obtained from CD melting experiment.

Sequences	Free(Tm)(°C)	Complex(Tm)(°C)	ΔTm(°C)
**BCL2**	57.1	73.3	16.2
**KRAS**	67.5	72.1	4.6
**VEGFA**	67.3	70.6	3.3

Table S4: Thermodynamic parameter derived from ITC experiment.

Parameters	BCL2	KRAS	VEGFA
**K_a(M^{-1})**	1.67×10^6	1.15×10^6	1.02×10^6
**K_D(μM)**	0.6	0.87	0.98
**ΔH(kcal.mol^{-1})**	-4.34×10^4	-4.14×10^4	-7.48×10^4
**ΔS(cal.mol^{-1}.deg^{-1})**	-119	-132	-111
**ΔG(kcal.mol^{-1})**	-8.5	-8.3	-8.2
**Binding Stiochiometry(n)**	0.32	0.30	0.29

Table S5: Primer used in RT-PCR.

Gene name	Gene ID	Transcript ID	Primers	Sequence	Orientation	Amplicon Size	Tm
KRAS	3845	NM_0049853	hKRAS-F	GACGAATATGATCCAACAATAGAGGATTC	Forward	244	57.3
			hKRAS-R	TAGGTACATCTTCAGAGTCCTTAACTC	Reverse		56.7
VEGFA	7422	NM_003376	hVEGFA-F	GGGCAGAATCATCACGAAGTGGTG	Forward	216	59.1
			hVEGFA-R	CTGCATGGTGATGTTGGACTCCTCA	Reverse		59.3
BCL2	596	NM_0006332	hBCL2-F	GTCATGTGTGTGGAGAGCGTCAACC	Forward	211	61
			hBCL2-R	CCAGGGCCAAACTGAGCAGAGTC	Reverse		60.6
GAPDH	2597	NM_002046	hGAPDH-F	GATGCTGGCGCTGAGTACGTCGTG	Forward	282	62.5
			hGAPDH-F	AGTGATGGCATGGACTGTGGTCATGAG	Reverse		61.3

Table S6: Details of the primer sequences for cloning.The 'Promoter+GQ' constructs are created by using wild-type (wt) primers, which contain KpnI and HindIII restriction sites. The restriction sites are underlined by blue and brown respectively. These constructs harbour the quadruplex forming motifs within the promoter regions of *BCL-2*, *VEGF-A*, and *KRAS*. 'GQ-null'corresponds to the inserts, which lack the quadruplex forming sequences.Mutagenic primers are used in first-round PCR reactions and wild-type primers in the final extension of one-step overlap-PCR experiments while creating these GQ-null constructs.The primer size, annealing temperature and construct size are listed.

		Primer sequences for reporter luciferase constructs (5′-3′)[†]	Size (bp)	Annealing temperature (^{0}C)	Construct size (bp)
VEGF-A	Promoter+GQ	**VEGF-A wt FP**: CGACCT**GGTACC**GAGCGAGCAGCGTCTTCGAGAGTGAGGACG	42	60.2	582
		**VEGG-A wt RP**: CTCGTC**AAGCTT**CAGCGCCACGACCTCCGAGCTAC	35	60.1	
	GQ-null	**VEGF-A mut FP:** CGCCTGTCCCCGCCCCCCCCATGCGCCCCCCCCTTTTTTTTTTAAAAG	48	67.2	546
		**VEGF-A mut RP:** CTTTTAAAAAAAAAAGGGGGGGGCGCATGGGGGGGGCGGGGACAGGCG	48	67.3	
BCL-2	Promoter+GQ	**BCL-2 wt FP:** CGACCT**GGTACC**CGCCCCCCTCCGCACTCCGTCGTC	35	60.2	759
		**BCL-2 wt RP:** CTCGTC**AAGCTT**GGAGCCCCGGCACCTTCGCTGGC	36	60.4	
	GQ-null	**BCL-2 mut FP:** GTAAGAGGACAGGCACCACAGCCCCCCCGCCCCTCCGCGCCGCC	43	67.5	723
		**BCL-2 mut RP:** GGCGGCGCGGAGGGGCGGGGGGGCTGTGGTGCCTGTCCTCTTAC	43	67.5	
KRAS	Promoter+GQ	**KRAS wt FP:** CGACCT**GGTACC**GCGATCAGACAGCCCCGGTGTGGGAAATC	36	67	605
		**KRAS wt RP:** CTCGTC**AAGCTT**GCTCGCTCCCAGTCCGAAATGGCG	35	67	
	GQ-null	**KRAS mut FP:** CTTCTCCCCGCCGGCGCTCGCTGCAGCCGCTCCCTCTCGTACG	44	67.9	583
		**KRAS mut RP:** CGTACGAGAGGGAGCGGCTGCAGCGAGCGCCGGCGGGGAGAAG	44	67.9	

[†]All the primers for cloning are obtained from Biobharati India Pvt. Ltd.

*All the mutant constructs are created over corresponding Promoter+GQ template and cloned into the KpnI and HindIII sites of pGL4.72[*hRlucCP*] luciferase vector.

GGTACC: KpnI restriction site
AAGCTT: HindIII restriction site

FP (Forward primer) and RP (Reverse primer)

Table S7: Thermodynamic parameter calculated from MMPBSA method for BCL2-Chelerythrine.

Parameters	1:1 Complex(3'-end face)	1:2 Complex(loop bases)
ΔE_{elec}	-559.49±5.59	-487.72±5.92
ΔE_{vdw}	-54.39±1.97	-41.92±1.92
ΔE_{mm}	-613.88±3.62	-529.63±4.01
ΔG_{PB}	607.11±5.16	507.61±5.66
ΔG_{np}	-33.75±0.09	-10.51±0.07
ΔG_{solv}	573.36±5.25	497.11±5.59
ΔG_{total}	-40.52±1.64	-32.59±1.58
**-TΔS**	-16.39±0.0	-18.64±0.0
ΔG_{bind}	-24.12±1.64	-13.94±1.58

Table S8: Thermodynamic parameter calculated from MMPBSA method for VEGFA-Chelerythrine complex.

Parameters	1:1 Complex(5'-end face)	1:2 Complex(3'-end face)
ΔE_{elec}	-488.01±7.1	-550.57±7.0
ΔE_{vdw}	-47.78±2.1	-47.68±2.3
ΔE_{mm}	-535.79±5.0	-598.25±4.7
ΔG_{PB}	536.69±6.44	613.06±8.3
ΔG_{np}	-35.90±0.53	-47.61±1.6
ΔG_{solv}	500.78±6.97	565.45±6.7
ΔG_{total}	-35±1.97	-32.8±1.9
**-TΔS**	-12.89±0.0	-15.24±0.0
ΔG_{bind}	-22.10±1.97	-17.56±1.94

Supplementary Information others

Cloning of wild-type KRAS promoter having quadruplex forming motif into pGL4.72 (KpnI - HindIII)

KRAS-ENST00000311936

gcgatcagacagccccggtgtgggaaatcgtccgcccggtctccctaagtccccgaagtcgcctccca
cttttggtgactgcttgtttatttacatgcagtcaatgatagtaaatggatgcgcgccagtataggcc
gaccctgagggtggcggggtgctcttcgcagcttctctgtggagaccggtcagcggggcggcgtggcc
gctcgcggcgtctccctggtggcatccgcacagcccgccgcggtccggtcccgctccgggtcagaatt
ggcggctgcggggacagccttgcggctaggcagggggcgggccgccgcgtgggtccggcagtccctcc
tcccgccaaggcgccgcccagacccgctctccagccggcccggctcgccaccctagaccgccccagcc
accccttcctccgccggcccggcccccgctcctcccccgccggcccggcccggcccccctccttctccc
cgccggcgctcgctg**cctcccccctcttccctcttcccacaccgccct**cagccgctccctctcgtacgc
ccgtctgaagaagaatcgagcgcggaacgcatcgatagctctgccctctgcggccgcccggccccgaa
ctcatcggtgtgctcggagctcgatttTCCTAGGCGGCGGCCGCGGCGGCGGAGGCAGCAGCGGCGGC
GGCAGTGGCGGCGGCGAAGGTGGCGGCGGCTCGGCCAGTACTCCCGGCCCCCGCCATTTCGGACTGGG
AGCGAGC

(Bold and underlined sequence is the complimentary strand of G-quadruplex motif in KRAS. In the mutant KRAS (GQ-null) this part had been deleted and then cloned into pGL4.72 vector)

Sequence of the constructs cloned into pGL4.72 vector:

>1st_BASE_2366059_Wt__KRS_RV_Primer_3
GGGATCTGTGCAGACTTTCTCTGGCCTAACTGGCCGGTACCGCGATCAGACAGCCCCGGTGTGGGAAATCG
TCCGCCCGGTCTCCCTAAGTCCCCGAAGTCGCCTCCCACTTTTGGTGACTGCTTGTTTATTTACATGCAGT
CAATGATAGTAAATGGATGCGCGCCAGTATAGGCCGACCCTGAGGGTGGCGGGGTGCTCTTCGCAGCTTCT
CTGTGGAGACCGGTCAGCGGGGCGGCGTGGCCGCTCGCGGCGTCTCCCTGGTGGCATCCGCACAGCCCGCC
GCGGTCCGGTCCCGCTCCGGGTCAGAATTGGCGGCTGCGGGGACAGCCTTGCGGCTAGGCAGGGGGCGGGC
CGCCGCGTGGGTCCGGCAGTCCCTCCTCCCGCCAAGGCGCCGCCCAGACCCGCTCTCCAGCCGGCCCGGCT
CGCCACCCTAGACCGCCCCAGCCACCCCTTCCTCCGCCGGCCCGGCCCCCGCTCCTCCCCCGCCGGCCCGG
CCCGGCCCCCTCCTTCTCCCCGCCGGCGCTCGCTGCCTCCCCCCTCTTCCCTCTTCCCACACCGCCCTCAGC
CGCTCCCTCTCGTACGCCCGTCTGAAGAAGAATCGAGCGCGGAACGCATCGATAGCTCTGCCCTCTGCGGC
CGCCCGGCCCCGAACTCATCGGTGTGCTCGGAGCTCGATTTTCCTAGGCGGCGGCCGCGGCGGCGGAGGCA
GCAGCGGCGGCGGCGGTGGCGGCGGCGAAGGTGGCGGCGGCTCGGCCAGTACTCCCGGCCCCCGCCATTTC
GGACTGGGAGCGAGCAAGCTTGGCAATCCGGTACTGTTGGTAAAGCCACCATGGCTTCCAAGGTGTACGAC
CCCGAGCAACGCAAACGCATGATCACTGGGCCTCAGTGGTGGGCTCGCTGCAAGCAAATGAACGTGCTGGA
CTCCTTCATCAACTACTATGATTCCGAGAAGCACGCCGAGAACGCCGTGATTTTTCTGCATGGTAACGCTG
CCTCCAGCTACCTGTGGGAGGCACGTCCTGGCCTCACATCGAGGCCCTGGGCTAGATGCATCATCCCCTAA
TCTGATCCG

The grey highlighted sequence is the wild-type KRAS promoter which harbours the quadruplex motif. Blue and orange underlined six-nucleotide long part corresponds to the KpnI and HindIII restriction sites.

Wild-type KRAS (Promoter+GQ) sequence alignment:

```
CLUSTAL multiple sequence alignment by MUSCLE (3.8)


Clone           GCGATCAGACAGCCCCGGTGTGGGAAATCGTCCGCCCGGTCTCCCTAAGTCCCCGAAGTC
Wt              GCGATCAGACAGCCCCGGTGTGGGAAATCGTCCGCCCGGTCTCCCTAAGTCCCCGAAGTC
                ************************************************************

Clone           GCCTCCCACTTTTGGTGACTGCTTGTTTATTTACATGCAGTCAATGATAGTAAATGGATG
Wt              GCCTCCCACTTTTGGTGACTGCTTGTTTATTTACATGCAGTCAATGATAGTAAATGGATG
                ************************************************************

Clone           CGCGCCAGTATAGGCCGACCCTGAGGGTGGCGGGGTGCTCTTCGCAGCTTCTCTGTGGAG
Wt              CGCGCCAGTATAGGCCGACCCTGAGGGTGGCGGGGTGCTCTTCGCAGCTTCTCTGTGGAG
                ************************************************************

Clone           ACCGGTCAGCGGGGCGGCGTGGCCGCTCGCGGCGTCTCCCTGGTGGCATCCGCACAGCCC
Wt              ACCGGTCAGCGGGGCGGCGTGGCCGCTCGCGGCGTCTCCCTGGTGGCATCCGCACAGCCC
                ************************************************************

Clone           GCCGCGGTCCGGTCCCGCTCCGGGTCAGAATTGGCGGCTGCGGGGACAGCCTTGCGGCTA
Wt              GCCGCGGTCCGGTCCCGCTCCGGGTCAGAATTGGCGGCTGCGGGGACAGCCTTGCGGCTA
                ************************************************************

Clone           GGCAGGGGGCGGGCCGCCGCGTGGGTCCGGCAGTCCCTCCTCCCGCCAAGGCGCCGCCCA
Wt              GGCAGGGGGCGGGCCGCCGCGTGGGTCCGGCAGTCCCTCCTCCCGCCAAGGCGCCGCCCA
                ************************************************************

Clone           GACCCGCTCTCCAGCCGGCCCGGCTCGCCACCCTAGACCGCCCCAGCCACCCCTTCCTCC
Wt              GACCCGCTCTCCAGCCGGCCCGGCTCGCCACCCTAGACCGCCCCAGCCACCCCTTCCTCC
                ************************************************************

Clone           GCCGGCCCGGCCCCCGCTCCTCCCCCGCCGGCCCGGCCCGGCCCCCTCCTTCTCCCCGCC
Wt              GCCGGCCCGGCCCCCGCTCCTCCCCCGCCGGCCCGGCCCGGCCCCCTCCTTCTCCCCGCC
                ************************************************************

Clone           GGCGCTCGCTGCCTCCCCCTCTTCCCTCTTCCCACACCGCCCTCAGCCGCTCCCTCTCGT
Wt              GGCGCTCGCTGCCTCCCCCTCTTCCCTCTTCCCACACCGCCCTCAGCCGCTCCCTCTCGT
                ************************************************************

Clone           ACGCCCGTCTGAAGAAGAATCGAGCGCGGAACGCATCGATAGCTCTGCCCTCTGCGGCCG
Wt              ACGCCCGTCTGAAGAAGAATCGAGCGCGGAACGCATCGATAGCTCTGCCCTCTGCGGCCG
                ************************************************************

Clone           CCCGGCCCCGAACTCATCGGTGTGCTCGGAGCTCGATTTTCCTAGGCGGCGGCCGCGGCG
Wt              CCCGGCCCCGAACTCATCGGTGTGCTCGGAGCTCGATTTTCCTAGGCGGCGGCCGCGGCG
                ************************************************************

Clone           GCGGAGGCAGCAGCGGCGGCGGCGGTGGCGGCGGCGAAGGTGGCGGCGGCTCGGCCAGTA
Wt              GCGGAGGCAGCAGCGGCGGCGGCAGTGGCGGCGGCGAAGGTGGCGGCGGCTCGGCCAGTA
                *********************** ************************************

Clone           CTCCCGGCCCCCGCCATTTCGGACTGGGAGCGAGC
Wt              CTCCCGGCCCCCGCCATTTCGGACTGGGAGCGAGC
                ***********************************
```

Cloning of mutant KRAS promoter withoutquadruplex forming motif into pGL4.72 (KpnI - HindIII)

>1st_BASE_2391102_Mut__KRS1_RV_Primer_3

```
GGGGTTTGGGCCGACATTTCTCTGGCCTAACTGGCCGGTGCCGCGATGAGACAGCCCCGGTGTGGGAAATC
GTCCGCCCGGTCGCCCTAAGTCCCCGAAGTCGCCTCCCACTTTTGGTGACTGCTTGTTTATTTATATGCAT
TCAATGATACTAATTGGATGCGCGCCAGTATAGGCCGACCCTGAGGGTGGCGGGGTGCTCTTCGCAGCTTC
TCTGTGGAGACCGGTCAGCGGGGCGGCGTGGCCGCTCGCGGCGTCTCCCTGGTGGCATCCGCACAGCCCGC
CGCGGTCCGGTCCCGCTCCGGGTCAGAATTGGCGGCTGCGGGGACAGCCTTGCGGCTAGGCAGGGGGCGGG
CCGCCGCGTGGGTCCGGCAGTCCCTCCTCCCGCCAAGGCGCCGCCCAGACCCGCTCTCCAGCCGGCCCGGC
TCGCCACCCTAGACCGCCCCAGCCACCCCTTCCTCCGCCGGCCCGGCCCCCGCTCCTCCCCCGCCGGCCCG
GCCCGGCCCCCTCCTTCTCCCCGCCGGCGCTCGCTGCAGCCGCTCCCTCTCGTACGCCCGTCTGAAGAAGA
ATCGAGCGCGGAACGCATCGATAGCTCTGCCCTCTGCGGCCGCCCGGCCCCGAACTCATCGGTGTGCTCGG
AGCTCGATTTTCCTAGGCGGCGGCCGCGGCGGCGGAGGCAGCAGCGGCGGCGGCGGTGGCGGCGGCGAAGG
TGGCGGCGGCTCGGCCAGTACTCCCGGCCCCCGCCATTTCGGACTGGGAGCGAGCAAGCTTGGCAATCCGG
TACTGTTGGTAAAGCCACCATGGCTTCCAAGGTGTACGACCCCGAGCAACGCAAACGCATGATCACTGGGC
CTCAGTGGTGGGCTCGCTGCAAGCAAATGAACGTGCTGGACTCCTTCATCAACTACTATGATTCCGAGAAG
CACGCCAAGAACGCCGTGATTTTTCTGCATGGTAACGCTGCCTCCAGCTACCTGTGGAAGGCACGTCGTGG
CCTCACATCGAACCCCGGGGCTAGATGCATCATCCCCTGATCTGATCGGAATGGGGTAAGTCCCGGCAAGA
GCGGGGAATGGGTTCATATCGCCCTCCTGGAATCACTACCAAGTACCCTCACCGGCTTGGGTTCGAACTTG
CTGAAACCTTTCAAAAGAAAAATCATCCTTTGTGGGGCCACGAACTGGGGGGGGCTTGGTCTGGGCCTTTT
TCCTTAATTCCTAACAAGGCACCAAGGACAAAGATTAAAGGGCCATTCGTTCCATGGCTGAAAAAGTGTCC
TGGGAAACCGTAATCCCAAATCCTTGGGGAACAAAATGGGCCCTTAACATTCAAAAGGAAGGATAAATCCC
CCCCTGTTTACAAAAAAGCAAAAAAAAAGGGCCCAAAAAAAAGAATGGTTCGG
```

Sequence alignment with wild-type KRAS

```
CLUSTAL multiple sequence alignment by MUSCLE (3.8)


wt              GCGATCAGACAGCCCCGGTGTGGGAAATCGTCCGCCCGGTCTCCCTAAGTCCCCGAAGTC
Mut             GCGATGAGACAGCCCCGGTGTGGGAAATCGTCCGCCCGGTCGCCCTAAGTCCCCGAAGTC
                ***** *********************************** ******************

wt              GCCTCCCACTTTTGGTGACTGCTTGTTTATTTACATGCAGTCAATGATAGTAAATGGATG
Mut             GCCTCCCACTTTTGGTGACTGCTTGTTTATTTATATGCATTCAATGATACTAATTGGATG
                ********************************* ***** ********* *** ******

wt              CGCGCCAGTATAGGCCGACCCTGAGGGTGGCGGGGTGCTCTTCGCAGCTTCTCTGTGGAG
Mut             CGCGCCAGTATAGGCCGACCCTGAGGGTGGCGGGGTGCTCTTCGCAGCTTCTCTGTGGAG
                ************************************************************

wt              ACCGGTCAGCGGGGCGGCGTGGCCGCTCGCGGCGTCTCCCTGGTGGCATCCGCACAGCCC
Mut             ACCGGTCAGCGGGGCGGCGTGGCCGCTCGCGGCGTCTCCCTGGTGGCATCCGCACAGCCC
                ************************************************************

wt              GCCGCGGTCCGGTCCCGCTCCGGGTCAGAATTGGCGGCTGCGGGGACAGCCTTGCGGCTA
Mut             GCCGCGGTCCGGTCCCGCTCCGGGTCAGAATTGGCGGCTGCGGGGACAGCCTTGCGGCTA
                ************************************************************
```

```
wt          GGCAGGGGGCGGGCCGCCGCGTGGGTCCGGCAGTCCCTCCTCCCGCCAAGGCGCCGCCCA
Mut         GGCAGGGGGCGGGCCGCCGCGTGGGTCCGGCAGTCCCTCCTCCCGCCAAGGCGCCGCCCA
            ************************************************************

wt          GACCCGCTCTCCAGCCGGCCCGGCTCGCCACCCTAGACCGCCCCAGCCACCCCTTCCTCC
Mut         GACCCGCTCTCCAGCCGGCCCGGCTCGCCACCCTAGACCGCCCCAGCCACCCCTTCCTCC
            ************************************************************

wt          GCCGGCCCGGCCCCCGCTCCTCCCCCGCCGGCCCGGCCCGGCCCCCTCCTTCTCCCCGCC
Mut         GCCGGCCCGGCCCCCGCTCCTCCCCCGCCGGCCCGGCCCGGCCCCCTCCTTCTCCCCGCC
            ************************************************************

wt          GGCGCTCGCTGCCTCCCCCTCTTCCCTCTTCCCACACCGCCCTCAGCCGCTCCCTCTCGT
Mut         GGCGCTCGCTG--------------------------------CAGCCGCTCCCTCTCGT
            ***********                                *****************

wt          ACGCCCGTCTGAAGAAGAATCGAGCGCGGAACGCATCGATAGCTCTGCCCTCTGCGGCCG
Mut         ACGCCCGTCTGAAGAAGAATCGAGCGCGGAACGCATCGATAGCTCTGCCCTCTGCGGCCG
            ************************************************************

wt          CCCGGCCCCGAACTCATCGGTGTGCTCGGAGCTCGATTTTCCTAGGCGGCGGCCGCGGCG
Mut         CCCGGCCCCGAACTCATCGGTGTGCTCGGAGCTCGATTTTCCTAGGCGGCGGCCGCGGCG
            ************************************************************

wt          GCGGAGGCAGCAGCGGCGGCGGCGGTGGCGGCGGCGAAGGTGGCGGCGGCTCGGCCAGTA
Mut         GCGGAGGCAGCAGCGGCGGCGGCGGTGGCGGCGGCGAAGGTGGCGGCGGCTCGGCCAGTA
            ************************************************************

wt          CTCCCGGCCCCCGCCATTTCGGACTGGGAGCGAGC
Mut         CTCCCGGCCCCCGCCATTTCGGACTGGGAGCGAGC
            ***********************************
```

Cloning of wild-type BCL2 promoter having quadruplex forming motif into pGL4.72 (KpnI - HindIII)

BCL-2ENST00000398117

cgcccccctccgcactccgtcgtccgcccggcccggccgcgtgcggttccccgggagcccccaccccg
tcgcggaccccagcgaccaccaagtccgcacgcggcctgccgcaggcctgagcagaaggccccgcgca
cacccaccgcgccgcggccgcgcgggaggcctgtgccgcccgcgccacccactggccgggccccgcgg
gcgcagcggagcgggcgggtggccggcccggacgcgccctccccggccgcggccccgcgcgccatgtg
cccccggcgggacgcgccactcccgggcctgccgcggcgcctttaacccgggccagggagcggggcgg
agggggcggtcgggtggctcagaggagggctctttctttcttctttttttgaatgaaccgtgtgacgt
tacgcacaggaaaccggtcgggctgtgcagagaatgaagtaagaggacaggcaccacagcccc**gctcc**
cgccccttcctcccGCGCCCGCCCCTCCGCGCCGCCTGCCCGCCCGCCCGCCGCGCTCCCGCCCGCC
GCTCTCCGTGGCCCCGCCGCGCTGCCGCCGCCGCCGCTGCCAGCGAAGGTGCCGGGGCTCC

(Bold and underlined sequence is the complimentary strand of G-quadruplex motif in BCL2. In the mutant BCL2 (GQ-null) this part had been deleted and then cloned into pGL4.72 vector)

Sequence file:

>1st_BASE_2391104_BCL2_RV_Primer_3

GGCGGGGCGGGCCGACTTTCTCTGGCCTAACTGGCCGGTACCCGCCCCCCTCCGCACTCCGTCGTCCGCCC
GGCCCGGCCGCGTGCGGTTCCCCGGGAGCCCCCACCCCGTCGCGGACCCCAGCGACCACCAAGTCCGCACG
CGGCCTGCCGCAGGCCTGAGCAGAAGGCCCCGCGCACACCCACCGCGCCGCGGCCGCGCGGGAGGCCTGTG
CCGCCCGCGCCACCCACTGGCCGGGCCCCGCGGGCGCAGCGGAGCGGGCGGGTGGCCGGCCCGGACGCGCC

CTCCCCGGCCGCGGCCCCGCGCGCCATGTGCCCCCGGCGGGACGCGCCACTCCCGGGCCTGCCGCGGCGCC
TTTAACCCGGGCCAGGGAGCGGGGCGGAGGGGGCGGTCGGGTGGCTCAGAGGAGGGCTCTTTCTTTCTTCT
TTTTTTGAATGAACCGTGTGACGTTACGCACAGGAAACCGGTCGGGCTGTGCAGAGAATGAAGTAAGAGGA
CAGGCACCACAGCCCCGCTCCCGCCCCCTTCCTCCCGCGCCCGCCCCTCCGCGCCGCCTGCCCGCCCGCCC
GCCGCGCTCCCGCCCGCCGCTCTCCGTGGCCCCGCCGCGCTGCCGCCGCCGCCGCTGCCAGCGAAGGTGCC
GGGGCTCCAAGCTTGGCAATCCGGTACTGTTGGTAAAGCCACCATGGCTTCCAAGGTGTACGACCCCGAGC
AACGCAAACGCATGATCACTGGGCCTCAGTGGTGGGCTCGCTGCAAGCAAATGAACGTGCTGGACTCCTTC
ATCAACTACTATGATTCCGAGAAGCACGCCGAGAACGCCGTGATTTTTCTGCATGGTAACGCTGCCTCCAG
CTACCTGTGGAGGCACGTCGTGCCTCACATCGAGCCCGTGGCTAGATGCATCATCCCTGATCTGATCGGAA
TGGTTAAGTCCGGTAAGAGCGGTAATGGCTCATATCGCCTCTTGTATCACTACAAGTTACCTCATCGTCTT
GGTTCGAGCTTGCTGAATCCTTCCAAAGAAAATCATCTTTGCTGGTCACTTACTGGGTGGGCTTGCTTGGG
TCTTTTATTAATTCCTACTTATCACCTATATATAGTATCAAGGTCCT

The grey highlighted sequence is the wild-type BCL2 promoter which harbours the quadruplex motif. Blue and orange underlined six-nucleotide long part corresponds to the KpnI and HindIII restriction sites.

Wild-type BCL 2 (Promoter+GQ) sequence alignment:

```
CLUSTAL multiple sequence alignment by MUSCLE (3.8)


wt              CGCCCCCCTCCGCACTCCGTCGTCCGCCCGGCCCGGCCGCGTGCGGTTCCCCGGGAGCCC
clone           CGCCCCCCTCCGCACTCCGTCGTCCGCCCGGCCCGGCCGCGTGCGGTTCCCCGGGAGCCC
                ************************************************************

wt              CCACCCCGTCGCGGACCCCAGCGACCACCAAGTCCGCACGCGGCCTGCCGCAGGCCTGAG
clone           CCACCCCGTCGCGGACCCCAGCGACCACCAAGTCCGCACGCGGCCTGCCGCAGGCCTGAG
                ************************************************************

wt              CAGAAGGCCCCGCGCACACCCACCGCGCCGCGGCCGCGCGGGAGGCCTGTGCCGCCCGCG
clone           CAGAAGGCCCCGCGCACACCCACCGCGCCGCGGCCGCGCGGGAGGCCTGTGCCGCCCGCG
                ************************************************************

wt              CCACCCACTGGCCGGGCCCCGCGGGCGCAGCGGAGCGGGCGGGTGGCCGGCCCGGACGCG
clone           CCACCCACTGGCCGGGCCCCGCGGGCGCAGCGGAGCGGGCGGGTGGCCGGCCCGGACGCG
                ************************************************************

wt              CCCTCCCCGGCCGCGGCCCCGCGCGCCATGTGCCCCCGGCGGGACGCGCCACTCCCGGGC
clone           CCCTCCCCGGCCGCGGCCCCGCGCGCCATGTGCCCCCGGCGGGACGCGCCACTCCCGGGC
                ************************************************************

wt              CTGCCGCGGCGCCTTTAACCCGGGCCAGGGAGCGGGGCGGAGGGGGCGGTCGGGTGGCTC
clone           CTGCCGCGGCGCCTTTAACCCGGGCCAGGGAGCGGGGCGGAGGGGGCGGTCGGGTGGCTC
                ************************************************************

wt              AGAGGAGGGCTCTTTCTTTCTTCTTTTTTTGAATGAACCGTGTGACGTTACGCACAGGAA
clone           AGAGGAGGGCTCTTTCTTTCTTCTTTTTTTGAATGAACCGTGTGACGTTACGCACAGGAA
                ************************************************************

wt              ACCGGTCGGGCTGTGCAGAGAATGAAGTAAGAGGACAGGCACCACAGCCCCGCTCCCGCC
clone           ACCGGTCGGGCTGTGCAGAGAATGAAGTAAGAGGACAGGCACCACAGCCCCGCTCCCGCC
                ************************************************************

wt              CCCTTCCTCCCGCGCCCGCCCCTCCGCGCCGCCTGCCCGCCCGCCCGCCGCGCTCCCGCC
clone           CCCTTCCTCCCGCGCCCGCCCCTCCGCGCCGCCTGCCCGCCCGCCCGCCGCGCTCCCGCC
                ************************************************************

wt              CGCCGCTCTCCGTGGCCCCGCCGCGCTGCCGCCGCCGCCGCTGCCAGCGAAGGTGCCGGG
clone           CGCCGCTCTCCGTGGCCCCGCCGCGCTGCCGCCGCCGCCGCTGCCAGCGAAGGTGCCGGG
                ************************************************************

wt              GCTCC
clone           GCTCC
                *****
```

Cloning of mutant BCL2 promoter without quadruplex forming motif into pGL4.72 (KpnI - HindIII)

```
>1st_BASE_2428613_M__BCL2__1__RV_Primer_3
GGAAGGTTAGGGCAGACTTTCTCTGGCCTACTGGCCGGTACCCGCCCCCCTCCGCACTCCGTCGTCCGCCC
GGCCCGGCCGCGTGCGGTTCCCCGGGAGCCCCCACCCCGTCGCGGACCCCAGCGACCACCAAGTCCGCACG
CGGCCTGCCGCAGGCCTGAGCAGAAGGCCCCGCGCACACCCACCGCGCCGCGGCCGCGCGGGAGGCCTGTG
CCGCCCGCGCCACCCACTGGCCGGGCCCCGCGGGCGCAGCGGAGCGGGCGGGTGGCCGGCCCGGACGCGCC
CTCCCCGGCCGCGGCCCCGCGCGCCATGTGCCCCCGGCGGGACGCGCCACTCCCGGGCCTGCCGCGGCGCC
TTTAACCCGGGCCAGGGAGCGGGGCGGAGGGGGCGGTCGGGTGGCTCAGAGGAGGGCTCTTTCTTTCTTCT
TTTTTTGAATGAACCGTGTGACGTTACGCACAGGAAACCGGTCGGGCTGTGCAGAGAATGAAGTAAGAGGA
CAGGCACCACAGCCCCCCCGCCCCTCCGCGCCGCCTGCCCGCCCGCCCGCCGCGCTCCCGCCCGCCGCTCT
CCGTGGCCCCGCCGCGCTGCCGCCGCCGCCGCTGCCAGCGAAGGTGCCGGGGCTCCAAGCTTGGCAATCCG
GTACTGTTGGTAAAGCCACCATGGCTTCCAAGGTGTACGACCCCGAGCAACGCAAACGCATGATCACTGGG
CCTCAGTGGTGGGCTCGCTGCAAGCAAATGAACGTGCTGGACTCCTTCATCAACTACTATGATTCCGAGAA
GCACGCCGAGAACGCCGTGATTTTTCTGCATGGTAACGCTGCCTCCAGCTACCTGTGGAGGCACGTCGTGC
CTCACATCGAGCCCGTGGCTAGATGCATCATCCCCGGATCTGATCGGAATGGGGTAAGTCCCGGCAAGAAG
CGGGAATGGGCTCATATCGCCCTCCTGGGATCACTACAAGTTACCTCACCGCCTTGGGTTCGAAGCTGCTG
AAACCTTCCAAAGAAAAATCATCTTTTGTGGGGCCACCGACTGGGGGGGGCTTGGTCTGGGCCTTTTCCCT
TACTTCCTACCAAGCACCAAGAACAAGAATCAAGGGCCATTCGTTCCATGGCTGAAAAGTGTCCGTGGAAC
GTGAATCCAGTTCCTGGGGAACGAAGGGGCCCTGACATTCCAAGGAAGGAAATTCCGCCCTGGATTCAAGA
AGCCGAAAAGAGGGCCAAGAATAAAAGGTTGGCCTTGGAAAAAAAAACTTTCCTTCCGTCCCAGAAACCAT
TGGCTTCCCGAAGCCAAAGAAATACTTGTTTGGAATAACTTGGGAACTCCTGGGAAGAGGATTCCCCTTGG
CCCCTAACCCTGGGGAATCCCCTTTTGCAAGGGGAAAATAAGCGGGCGGAAGGGATTTAAATACGGGGTCC
AATATTCCCTTTCCCTTCGTGGGCCTATC
```

The grey highlighted sequence is the mutant BCL2 promoter which lacks the quadruplex motif. Blue and orange underlined six-nucleotide long part corresponds to the KpnI and HindIII restriction sites.

Sequence alignment with wild-type*BCL-2:*

```
CLUSTAL multiple sequence alignment by MUSCLE (3.8)


wt              CGCCCCCCTCCGCACTCCGTCGTCCGCCCGGCCCGGCCGCGTGCGGTTCCCCGGGAGCCC
Mut1            CGCCCCCCTCCGCACTCCGTCGTCCGCCCGGCCCGGCCGCGTGCGGTTCCCCGGGAGCCC
                ************************************************************

wt              CCACCCCGTCGCGGACCCCAGCGACCACCAAGTCCGCACGCGGCCTGCCGCAGGCCTGAG
Mut1            CCACCCCGTCGCGGACCCCAGCGACCACCAAGTCCGCACGCGGCCTGCCGCAGGCCTGAG
                ************************************************************

wt              CAGAAGGCCCCGCGCACACCCACCGCGCCGCGGCCGCGCGGGAGGCCTGTGCCGCCCGCG
Mut1            CAGAAGGCCCCGCGCACACCCACCGCGCCGCGGCCGCGCGGGAGGCCTGTGCCGCCCGCG
                ************************************************************

wt              CCACCCACTGGCCGGGCCCCGCGGGCGCAGCGGAGCGGGCGGGTGGCCGGCCCGGACGCG
Mut1            CCACCCACTGGCCGGGCCCCGCGGGCGCAGCGGAGCGGGCGGGTGGCCGGCCCGGACGCG
                ************************************************************

wt              CCCTCCCCGGCCGCGGCCCCGCGCGCCATGTGCCCCCGGCGGGACGCGCCACTCCCGGGC
Mut1            CCCTCCCCGGCCGCGGCCCCGCGCGCCATGTGCCCCCGGCGGGACGCGCCACTCCCGGGC
                ************************************************************

wt              CTGCCGCGGCGCCTTTAACCCGGGCCAGGGAGCGGGGCGGAGGGGGCGGTCGGGTGGCTC
Mut1            CTGCCGCGGCGCCTTTAACCCGGGCCAGGGAGCGGGGCGGAGGGGGCGGTCGGGTGGCTC
                ************************************************************

wt              AGAGGAGGGCTCTTTCTTTCTTCTTTTTTTGAATGAACCGTGTGACGTTACGCACAGGAA
Mut1            AGAGGAGGGCTCTTTCTTTCTTCTTTTTTTGAATGAACCGTGTGACGTTACGCACAGGAA
                ************************************************************
```

```
wt              ACCGGTCGGGCTGTGCAGAGAATGAAGTAAGAGGACAGGCACCACAGCCCCGCTCCCGCC
Mut1            ACCGGTCGGGCTGTGCAGAGAATGAAGTAAGAGGACAGGCACCACAGCCCC---------
                ***************************************************

wt              CCCTTCCTCCCGCGCCCGCCCCTCCGCGCCGCCTGCCCGCCCGCCCGCCGCGCTCCCGCC
Mut1            --------------CCCGCCCCTCCGCGCCGCCTGCCCGCCCGCCCGCCGCGCTCCCGCC
                              **********************************************

wt              CGCCGCTCTCCGTGGCCCCGCCGCGCTGCCGCCGCCGCCGCTGCCAGCGAAGGTGCCGGG
Mut1            CGCCGCTCTCCGTGGCCCCGCCGCGCTGCCGCCGCCGCCGCTGCCAGCGAAGGTGCCGGG
                ************************************************************

wt              GCTCC
Mut1            GCTCC
                *****
```

Cloning of wt. VEGFA in pGL4.72 Using KpnI- HindIII

VEGFA-ENST00000372067:

Gagcgagcagcgtcttcgagagtgaggacgtgtgtgtctgtgtgggtgagtgagtgtgtgcgtgtggg
gttgagggcgttggagcggggagaaggccaggggtcactccaggattccaatagatctgtgtgtccct
ctccccacccgtccctgtccggctctccgccttcccctgccccccttcaatattcctagcaaagaggga
acggctctcaggccctgtccgcacgtaacctcactttcctgctccctcctcgccaatgccccgcgggc
gcgtgtctctggacagagtttccgggggcggatgggtaattttcaggctgtgaaccttggtgggggtc
gagcttccccttcattgcggcgggctgcgggccaggcttcactgagcgtccgcagagcccgggcccga
gccgcgtgtggaagggctgaggctcgcctgtccccgcccccc**ggggcgggccgggggcggggtcccgg**
cggggcggagccatgcgcccccccctttttttttaaaagtcggctggtagcggggaggatcgGGAGG
CTTGGGGCAGCCGGGTAGCTCGGAGGTCGTGGCGCTG

(Bold and underlined sequence is the G-quadruplex motif in VEGFA. In the mutant VEGFA (GQ-null) this part had been deleted and then cloned into pGL4.72 vector)

>1st_BASE_2362169_Wt__VEGF_RV_Primer_3

CCCCCCCTCCGGTGCCGACTTTCTCTGGCCTAACTGGCCGGTACCGAGCGAGCAGCGTCTTCGAGAGTGAG
GACGTGTGTGTCTGTGTGGGTGAGTGAGTGTGTGCGTGTGGGGTTGAGGGCGTTGGAGCGGGGAGAAGGCC
AGGGGTCACTCCAGGATTCCAATAGATCTGTGTGTCCCTCTCCCCACCCGTCCCTGTCCGGCTCTCCGCCT
TCCCCTGCCCCCCTTCAATATTCCTAGCAAAGAGGGAACGGCTCTCAGGCCCTGTCCGCACGTAACCTCACT
TTCCTGCTCCCTCCTCGCCAATGCCCCGCGGGCGCGTGTCTCTGGACAGAGTTTCCGGGGGCGGATGGGTA
ATTTTCAGGCTGTGAACCTTGGTGGGGGTCGAGCTTCCCCTTCATTGCGGCGGGCTGCGGGCCAGGCTTCA
CTGAGCGTCCGCAGAGCCCGGGCCCGAGCCGCGTGTGGAGGGGCTGAGGCTCGCCTGTCCCCGCCCCCCGG
GGCGGGCCGGGGGCGGGGTCCCGGCGGGGCGGAGCCATGCGCCCCCCCCTTTTTTTTTAAAAGTCGGCTG
GTAGCGGGGAGGATCGCGGAGGCTTGGGGCAGCCGGGTAGCTCGGAGGTCGTGGCGCTGAAGCTTGGCAAT
CCGGTACTGTTGGTAAAGCCACCATGGCTTCCAAGGTGTACGACCCCGAGCAACGCAAACGCATGATCACT
GGGCCTCAGTGGTGGGCTCGCTGCAAGCAAATGAACGTGCTGGACTCCTTCATCAACTACTATGATTCCGA
GAAGCACGCCGAGAACGCCGTGATTTTTCTGCATGGTAACGCTGCCTCCAGCTACCTGTGGAGGCACGTCG
TGCCTCACATCGAGCCCGTGGCTAGATGCATCATCCCTGATCTGATCGGAATGGGTAAGTCCGGCAAGAGC
GGGAATGGCTCATATCGCCTCCTGGATCACTACAAGTACCTCACCGCTTGGTTCGAGCTGCTGAACCTTCC

```
AAAGAAAATCATCTTTGTGGGCCACGACTGGGGGGGCTTGTCTGGGCCTTTCACTACTCCTTACGAGCACC
AAGAACAAGATCAAGGCCATCGTCCATGCTGAAGATTGTCCTGGAACGTGATCCGAATCCTGGGAACGAGT
GGCCTGAACTCCGAGGAGGATATCGCCCCTGATTAAAGAGCGAAAAAGGGCGAAAAAAATGGTGGCTTGGA
AAATAACTTCTTCCTCCAAAACCATGCTTCCCAAGCAAGATCATGGCGGAAACTGGGACCCTGAGGAATTC
GCTTGCCTACCTGGGAACCCTTTCAAGGGATAAGGGCGGAGGGTTAGAAGGGCTTACCCTTTCTTGGGTCA
```

The grey highlighted sequence is the wild-type VEGFA promoter which harbors the quadruplex motif. Blue and orange underlined six-nucleotide long part corresponds to the KpnI and HindIII restriction sites.

Wild-type VEGFA (Promoter+GQ) sequence alignment:

```
CLUSTAL multiple sequence alignment by MUSCLE (3.8)


VEGFA           GAGCGAGCAGCGTCTTCGAGAGTGAGGACGTGTGTGTCTGTGTGGGTGAGTGAGTGTGTG
clone           GAGCGAGCAGCGTCTTCGAGAGTGAGGACGTGTGTGTCTGTGTGGGTGAGTGAGTGTGTG
                ************************************************************

VEGFA           CGTGTGGGGTTGAGGGCGTTGGAGCGGGGAGAAGGCCAGGGGTCACTCCAGGATTCCAAT
clone           CGTGTGGGGTTGAGGGCGTTGGAGCGGGGAGAAGGCCAGGGGTCACTCCAGGATTCCAAT
                ************************************************************

VEGFA           AGATCTGTGTGTCCCTCTCCCCACCCGTCCCTGTCCGGCTCTCCGCCTTCCCCTGCCCCC
clone           AGATCTGTGTGTCCCTCTCCCCACCCGTCCCTGTCCGGCTCTCCGCCTTCCCCTGCCCCC
                ************************************************************

VEGFA           TTCAATATTCCTAGCAAAGAGGGAACGGCTCTCAGGCCCTGTCCGCACGTAACCTCACTT
clone           TTCAATATTCCTAGCAAAGAGGGAACGGCTCTCAGGCCCTGTCCGCACGTAACCTCACTT
                ************************************************************

VEGFA           TCCTGCTCCCTCCTCGCCAATGCCCCGCGGGCGCGTGTCTCTGGACAGAGTTTCCGGGGG
clone           TCCTGCTCCCTCCTCGCCAATGCCCCGCGGGCGCGTGTCTCTGGACAGAGTTTCCGGGGG
                ************************************************************

VEGFA           CGGATGGGTAATTTTCAGGCTGTGAACCTTGGTGGGGGTCGAGCTTCCCCTTCATTGCGG
clone           CGGATGGGTAATTTTCAGGCTGTGAACCTTGGTGGGGGTCGAGCTTCCCCTTCATTGCGG
                ************************************************************

VEGFA           CGGGCTGCGGGCCAGGCTTCACTGAGCGTCCGCAGAGCCCGGGCCCGAGCCGCGTGTGGA
clone           CGGGCTGCGGGCCAGGCTTCACTGAGCGTCCGCAGAGCCCGGGCCCGAGCCGCGTGTGGA
                ************************************************************

VEGFA           AGGGCTGAGGCTCGCCTGTCCCCGCCCCCCGGGGCGGGCCGGGGGCGGGGTCCCGGCGGG
clone           GGGGCTGAGGCTCGCCTGTCCCCGCCCCCCGGGGCGGGCCGGGGGCGGGGTCCCGGCGGG
                 ***********************************************************

VEGFA           GCGGAGCCATGCGCCCCCCCCTTTTTTTTTTAAAAGTCGGCTGGTAGCGGGGAGGATCGC
clone           GCGGAGCCATGCGCCCCCCCCTTTTTTTTTTAAAAGTCGGCTGGTAGCGGGGAGGATCGC
                ************************************************************

VEGFA           GGAGGCTTGGGGCAGCCGGGTAGCTCGGAGGTCGTGGCGCTG
clone           GGAGGCTTGGGGCAGCCGGGTAGCTCGGAGGTCGTGGCGCTG
                ******************************************
```

Cloning of mutant VEGFA promoter without quadruplex forming motif into pGL4.72 (KpnI - HindIII)

```
>1st_BASE_2362171_Mut__VEGF_2_RV_Primer_3
GCCCCAGTCCGTGCCGACTTTCTCTGGCCTAACTGGCCGGTACCGAGCGAGCAGCGTCTTCGAGAGTGAGG
ACGTGTGTGTCTGTGTGGGTGAGTGAGTGTGTGCGTGTGGGGTTGAGGGCGTTGGAGCGGGGAGAAGGCCA
GGGGTCACTCCAGGATTCCAATAGATCTGTGTGTCCCTCTCCCCACCCGTCCCTGTCCGGCTCTCCGCCTT
CCCCTGCCCCCTTCAATATTCCTAGCAAAGAGGGAACGGCTCTCAGGCCCTGTCCGCACGTAACCTCACTT
TCCTGCTCCCTCCTCGCCAATGCCCCGCGGGCGCGTGTCTCTGGACAGAGTTTCCGGGGGCGGATGGGTAA
TTTTCAGGCTGTGAACCTTGGTGGGGGTCGAGCTTCCCCTTCATTGCGGCGGGCTGCGGGCCAGGCTTCAC
TGAGCGTCCGCAGAGCCCGGGCCCGAGCCGCGTGTGGAGGGGCTGAGGCTCGCCTGTCCCCGCCCCCCCA
TGCGCCCCCCCCTTTTTTTTTTAAAAGTCGGCTGGTAGCGGGGAGGATCGCGGAGGCTTGGGGCAGCCGGG
TAGCTCGGAGGTCGTGGCGCTGAAGCTTGGCAATCCGGTACTGTTGGTAAAGCCACCATGGCTTCCAAGGT
GTACGACCCCGAGCAACGCAAACGCATGATCACTGGGCCTCAGTGGTGGGCTCGCTGCAAGCAAATGAACG
TGCTGGACTCCTTCATCAACTACTATGATTCCGAGAAGCACGCCGAGAACGCCGTGATTTTTCTGCATGGT
AACGCTGCCTCCAGCTACCTGTGGAGGCACGTCGTGCCTCACATCGAGCCCGTGGCTAGATGCATCATCCC
TGATCTGATCGGAATGGGTAAGTCCGGCAAGAGCGGGAATGGCTCATATCGCCTCCTGGATCACTACAAGT
ACCTCACCGCTTGGTTCGAGCTGCTGAACCTTCCAAAGAAAATCATCTTTGTGGGCCACGACTGGGGGGC
TTGTCTGGGCCTTTCACTACTCCTACGAGCACCAAGAACAAGATCAAGGGCCATCGTTCCATGCTTAAAAG
TGTCCTGGAACGTGATCCAATTCCTGGGAACGAATGGGCCTGACCTCCGAGGAAGGATATCCCCCCTGATC
AAGAACCGAAAAAGGGCGAAAAAAATGGTGGCTTGGAAAATAACTTTCTTCCTCCAAGACCATGCTTCCCA
AGCAAGAATCATGGCGGAAAACTGGGAGCCTGAAGGAATTCCCTTGCCTACCTGGGAAGCCATTCAAGGGA
AAAGGGCCGAGGTTTAGACGGGCCTAACCTCTTCCCTGGCCTCTCGGAGATTCCCCTTCCTTTTAAGGGGA
GGGCAGCCCCGGACTTTCTTCCCAAATTGGTCCGCAAATTAACCAAGCCTAACTCTGCCGGGCACCGGAAT
AAC
```

The grey highlighted sequence is the mutant VEGFA promoter which lacks the quadruplex motif. Blue and orange underlined six-nucleotide long part corresponds to the KpnI and HindIII restriction sites.

Sequence alignment with wild-type VEGFA*:*

```
CLUSTAL multiple sequence alignment by MUSCLE (3.8)


Mut             GAGCGAGCAGCGTCTTCGAGAGTGAGGACGTGTGTGTCTGTGTGGGTGAGTGAGTGTGTG
Wt              GAGCGAGCAGCGTCTTCGAGAGTGAGGACGTGTGTGTCTGTGTGGGTGAGTGAGTGTGTG
                ************************************************************

Mut             CGTGTGGGGTTGAGGGCGTTGGAGCGGGGAGAAGGCCAGGGGTCACTCCAGGATTCCAAT
Wt              CGTGTGGGGTTGAGGGCGTTGGAGCGGGGAGAAGGCCAGGGGTCACTCCAGGATTCCAAT
                ************************************************************

Mut             AGATCTGTGTGTCCCTCTCCCCACCCGTCCCTGTCCGGCTCTCCGCCTTCCCCTGCCCCC
Wt              AGATCTGTGTGTCCCTCTCCCCACCCGTCCCTGTCCGGCTCTCCGCCTTCCCCTGCCCCC
                ************************************************************

Mut             TTCAATATTCCTAGCAAAGAGGGAACGGCTCTCAGGCCCTGTCCGCACGTAACCTCACTT
Wt              TTCAATATTCCTAGCAAAGAGGGAACGGCTCTCAGGCCCTGTCCGCACGTAACCTCACTT
                ************************************************************

Mut             TCCTGCTCCCTCCTCGCCAATGCCCCGCGGGCGCGTGTCTCTGGACAGAGTTTCCGGGGG
Wt              TCCTGCTCCCTCCTCGCCAATGCCCCGCGGGCGCGTGTCTCTGGACAGAGTTTCCGGGGG
                ************************************************************

Mut             CGGATGGGTAATTTTCAGGCTGTGAACCTTGGTGGGGGTCGAGCTTCCCCTTCATTGCGG
Wt              CGGATGGGTAATTTTCAGGCTGTGAACCTTGGTGGGGGTCGAGCTTCCCCTTCATTGCGG
```

```
                 ************************************************************

Mut              CGGGCTGCGGGCCAGGCTTCACTGAGCGTCCGCAGAGCCCGGGCCCGAGCCGCGTGTGGA
Wt               CGGGCTGCGGGCCAGGCTTCACTGAGCGTCCGCAGAGCCCGGGCCCGAGCCGCGTGTGGA
                 ************************************************************

Mut              GGGGCTGAGGCTCGCCTGTCCCCGCCCCCC------------------------------
Wt               GGGGCTGAGGCTCGCCTGTCCCCGCCCCCCGGGGCGGGCCGGGGGCGGGGTCCCGGCGGG
                 ******************************

Mut              ------CCATGCGCCCCCCCCTTTTTTTTTTAAAAGTCGGCTGGTAGCGGGGAGGATCGC
Wt               GCGGAGCCATGCGCCCCCCCCTTTTTTTTTTAAAAGTCGGCTGGTAGCGGGGAGGATCGC
                       ******************************************************

Mut              GGAGGCTTGGGGCAGCCGGGTAGCTCGGAGGTCGTGGCGCTG
Wt               GGAGGCTTGGGGCAGCCGGGTAGCTCGGAGGTCGTGGCGCTG
                 ******************************************
```
